# Determination of the strong coupling constant ${\varvec{{\alpha _\mathrm{s} (m_\mathrm{Z})}}}$ in next-to-next-to-leading order QCD using H1 jet cross section measurements: H1 Collaboration

**DOI:** 10.1140/epjc/s10052-017-5314-7

**Published:** 2017-11-22

**Authors:** V. Andreev, A. Baghdasaryan, K. Begzsuren, A. Belousov, V. Bertone, A. Bolz, V. Boudry, G. Brandt, V. Brisson, D. Britzger, A. Buniatyan, A. Bylinkin, L. Bystritskaya, A. J. Campbell, K. B. Cantun Avila, K. Cerny, V. Chekelian, J. G. Contreras, J. Cvach, J. Currie, J. B. Dainton, K. Daum, C. Diaconu, M. Dobre, V. Dodonov, G. Eckerlin, S. Egli, E. Elsen, L. Favart, A. Fedotov, J. Feltesse, M. Fleischer, A. Fomenko, E. Gabathuler, J. Gayler, T. Gehrmann, S. Ghazaryan, L. Goerlich, N. Gogitidze, M. Gouzevitch, C. Grab, A. Grebenyuk, T. Greenshaw, G. Grindhammer, C. Gwenlan, D. Haidt, R. C. W. Henderson, J. Hladkỳ, D. Hoffmann, R. Horisberger, T. Hreus, F. Huber, A. Huss, M. Jacquet, X. Janssen, A. W. Jung, H. Jung, M. Kapichine, J. Katzy, C. Kiesling, M. Klein, C. Kleinwort, R. Kogler, P. Kostka, J. Kretzschmar, D. Krücker, K. Krüger, M. P. J. Landon, W. Lange, P. Laycock, A. Lebedev, S. Levonian, K. Lipka, B. List, J. List, B. Lobodzinski, E. Malinovski, H.-U. Martyn, S. J. Maxfield, A. Mehta, A. B. Meyer, H. Meyer, J. Meyer, S. Mikocki, A. Morozov, K. Müller, Th. Naumann, P. R. Newman, C. Niebuhr, J. Niehues, G. Nowak, J. E. Olsson, D. Ozerov, C. Pascaud, G. D. Patel, E. Perez, A. Petrukhin, I. Picuric, H. Pirumov, D. Pitzl, R. Plačakytė, R. Polifka, K. Rabbertz, V. Radescu, N. Raicevic, T. Ravdandorj, P. Reimer, E. Rizvi, P. Robmann, R. Roosen, A. Rostovtsev, M. Rotaru, D. Šálek, D. P. C. Sankey, M. Sauter, E. Sauvan, S. Schmitt, L. Schoeffel, A. Schöning, F. Sefkow, S. Shushkevich, Y. Soloviev, P. Sopicki, D. South, V. Spaskov, A. Specka, M. Steder, B. Stella, U. Straumann, M. R. Sutton, T. Sykora, P. D. Thompson, D. Traynor, P. Truöl, I. Tsakov, B. Tseepeldorj, A. Valkárová, C. Vallée, P. Van Mechelen, Y. Vazdik, D. Wegener, E. Wünsch, J. Žáček, Z. Zhang, R. Žlebčík, H. Zohrabyan, F. Zomer

**Affiliations:** 1 I. Physikalisches Institut der RWTH, Aachen, Germany; 2 School of Physics and Astronomy, University of Birmingham, Birmingham, UK; 3 Inter-University Institute for High Energies ULB-VUB, Brussels and Universiteit Antwerpen, Antwerp, Belgium; 4 Horia Hulubei National Institute for R&D in Physics and Nuclear Engineering (IFIN-HH), Bucharest, Romania; 5 STFC, Rutherford Appleton Laboratory, Didcot, Oxfordshire UK; 6 Institute of Nuclear Physics, Polish Academy of Sciences, 31342 Kraków, Poland; 7 Institut für Physik, TU Dortmund, Dortmund, Germany; 8 Joint Institute for Nuclear Research, Dubna, Russia; 9 Irfu/SPP, CE Saclay, Gif-sur-Yvette, France; 10 DESY, Hamburg, Germany; 11 Institut für Experimentalphysik, Universität Hamburg, Hamburg, Germany; 12 Physikalisches Institut, Universität Heidelberg, Heidelberg, Germany; 13 Department of Physics, University of Lancaster, Lancaster, UK; 14 Department of Physics, University of Liverpool, Liverpool, UK; 15 School of Physics and Astronomy, Queen Mary University of London, London, UK; 16 Aix Marseille Université, CNRS/IN2P3, CPPM UMR 7346, 13288 Marseille, France; 17 Departamento de Fisica Aplicada, CINVESTAV, Mérida, Yucatán Mexico; 18 Institute for Theoretical and Experimental Physics, Moscow, Russia; 19 Lebedev Physical Institute, Moscow, Russia; 20 Max-Planck-Institut für Physik, Munich, Germany; 21 LAL, Université Paris-Sud, CNRS/IN2P3, Orsay, France; 22 LLR, Ecole Polytechnique, CNRS/IN2P3, Palaiseau, France; 23 Faculty of Science, University of Montenegro, Podgorica, Montenegro; 24 Institute of Physics, Academy of Sciences of the Czech Republic, Prague, Czech Republic; 25 Faculty of Mathematics and Physics, Charles University, Prague, Czech Republic; 26 Dipartimento di Fisica, Università di Roma Tre and INFN Roma 3, Rome, Italy; 27 Institute for Nuclear Research and Nuclear Energy, Sofia, Bulgaria; 28 Institute of Physics and Technology of the Mongolian Academy of Sciences, Ulaanbaatar, Mongolia; 29 Paul Scherrer Institute, Villigen, Switzerland; 30 Fachbereich C, Universität Wuppertal, Wuppertal, Germany; 31 Yerevan Physics Institute, Yerevan, Armenia; 32 DESY, Zeuthen, Germany; 33 Institut für Teilchenphysik, ETH Zürich, Zurich, Switzerland; 34 Physik-Institut der Universität Zürich, Zurich, Switzerland; 35 IPNL, Université Claude Bernard Lyon 1, CNRS/IN2P3, Villeurbanne, France; 36 Skobeltsyn Institute of Nuclear Physics, Lomonosov Moscow State University, Moscow, Russia; 37 CERN, Geneva, Switzerland; 38 Ulaanbaatar University, Ulaanbaatar, Mongolia; 39 Department of Physics, University of Toronto, Toronto, ON M5S 1A7 Canada; 40 LAPP, Université de Savoie, CNRS/IN2P3, Annecy-le-Vieux, France; 41 II. Physikalisches Institut, Universität Göttingen, Göttingen, Germany; 42 Institute for Information Transmission Problems RAS, Moscow, Russia; 43 Moscow Institute of Physics and Technology, Dolgoprudny, Moscow Region Russian Federation; 44 Department of Physics, Oxford University, Oxford, UK; 45 Department of Physics and Astronomy, Purdue University, 525 Northwestern Ave, West Lafayette, IN 47907 USA; 46 Department of Physics and Astronomy, Vrije University, De Boelelaan 1081, Amsterdam, The Netherlands; 47 National Institute for Subatomic Physics (NIKHEF), Science Park 105, Amsterdam, The Netherlands; 48 Institute for Particle Physics Phenomenology, Ogden Centre for Fundamental Physics, Durham University, South Road, Durham, UK; 49 Karlsruher Institut für Technologie (KIT), Institut für Experimentelle Teilchenphysik (ETP), Wolfgang-Gaede-Str. 1, Karlsruhe, Germany; 50 Department of Physics and Astronomy, University of Sussex, Pevensey II, Brighton, UK

## Abstract

The strong coupling constant $\alpha _\mathrm{s}$ is determined from inclusive jet and dijet cross sections in neutral-current deep-inelastic *ep* scattering (DIS) measured at HERA by the H1 collaboration using next-to-next-to-leading order (NNLO) QCD predictions. The dependence of the NNLO predictions and of the resulting value of $\alpha _\mathrm{s} (m_\mathrm{Z})$ at the *Z*-boson mass $m_Z$ are studied as a function of the choice of the renormalisation and factorisation scales. Using inclusive jet and dijet data together, the strong coupling constant is determined to be $\alpha _\mathrm{s} (m_\mathrm{Z}) =0.1157\,(20)_\mathrm{exp}\,(29)_\mathrm{th}$. Complementary, $\alpha _\mathrm{s} (m_\mathrm{Z})$ is determined together with parton distribution functions of the proton (PDFs) from jet and inclusive DIS data measured by the H1 experiment. The value $\alpha _\mathrm{s} (m_\mathrm{Z}) =0.1142\,(28)_\mathrm{tot}$ obtained is consistent with the determination from jet data alone. The impact of the jet data on the PDFs is studied. The running of the strong coupling is tested at different values of the renormalisation scale and the results are found to be in agreement with expectations.

## Introduction

The strong coupling constant is one of the least well known parameters of the Standard Model of particle physics (SM) and a precise knowledge of this coupling is crucial for precision measurements, consistency tests of the SM and searches for physics beyond the SM. It has been determined in a large variety of processes and using different techniques [1, 2]. Jet production in the Breit frame [3] in neutral-current deep-inelastic *ep* scattering (NC DIS) is directly sensitive to the strong coupling and has a clean experimental signature with sizable cross sections. It is thus ideally suited for the precision determination of the strong coupling constant $\alpha _\mathrm{s} (m_\mathrm{Z})$ at the *Z*-boson mass $m_Z$.

Cross section predictions for inclusive jet and dijet production in NC DIS are obtained within the framework of perturbative QCD (pQCD) [4], where for the past 25 years only next-to-leading order (NLO) calculations have been available [5, 6]. Continuous developments enabled the advancement of these calculations [7–10], and next-to-next-to-leading order (NNLO) predictions for jet production in DIS [11, 12] and hadron-hadron collisions [13, 14] have become available recently. The theoretical uncertainties of the NNLO predictions are substantially reduced compared to those of the NLO predictions. It is observed [11, 12, 15] that the NNLO predictions and the current experimental data are of comparable precision for large parts of the measured phase space.

Measurements of inclusive jet and dijet cross sections in NC DIS have been performed at HERA by the H1 [15–24] and ZEUS [25–32] collaborations during different data taking periods and for different centre-of-mass energies. In general, the predictions in pQCD provide a good description of these data.

The strong coupling constant has been determined from jet cross sections in DIS at NLO accuracy [15, 17, 21–24, 27, 30, 33–35] and the precision of $\alpha _\mathrm{s} (m_\mathrm{Z})$ of these determinations is typically limited by the scale uncertainty of the NLO calculations. Only recently an $\alpha _\mathrm{s}$ determination was performed using inclusive jet cross sections, where NLO calculations have been supplemented with contributions beyond NLO in the threshold resummation formalism, and a moderate reduction of the scale uncertainty was achieved [36].

Measurements of jet production cross sections in processes other than NC DIS, such as photoproduction [37, 38] or in $e^+e^-$ [39–44], $p\bar{p}$ [45–47] and *pp* collisions [48–52], have also been employed for the determination of the strong coupling constant. The corresponding predictions were at NLO accuracy in most cases, possibly supplemented with 2-loop threshold corrections or matched with next-to-leading logarithmic approximations (NLLA). An exception are 3-jet observables in $e^+e^-$ collisions using predictions in NNLO accuracy [42], which are also matched to NLLA contributions [43, 44]. In contrast to variables such as hadronic event shape observables [53, 54] where only limited regions of the corresponding distributions are described by fixed order pQCD calculations, jet observables such as their transverse momenta typically are well described by such calculations over the full experimentally accessible range.

The presence of a proton in the initial state in lepton-hadron or hadron-hadron collisions complicates the determination of $\alpha _\mathrm{s} (m_\mathrm{Z})$ and therefore $\alpha _\mathrm{s} (m_\mathrm{Z})$ is often determined together with parton distribution functions of the proton (PDFs). Such simultaneous determinations of $\alpha _\mathrm{s} (m_\mathrm{Z})$ and PDFs were performed using jet cross sections in DIS [17, 55, 56] or jet cross sections at either the LHC or Tevatron [50, 52, 57–59]. However, the absence of full NNLO corrections for jet production cross sections limited the theoretical precision of these approaches.

This article presents the first determination of $\alpha _\mathrm{s} (m_\mathrm{Z})$ making use of the recent calculations of jet production at NNLO [11–14]. These calculations are also used in this paper for the first time for the determination of PDFs. The jet cross section calculations are performed using the program  [11, 12, 60].

Two strategies for the extraction of $\alpha _\mathrm{s} (m_\mathrm{Z})$ are investigated. First, described in Sect. 3, the value of $\alpha _\mathrm{s} (m_\mathrm{Z})$ is determined in NNLO from inclusive jet and dijet cross sections [15, 17, 21, 23, 24] using pre-determined PDFs as input. In a second approach described in Sect. 4, the value of $\alpha _\mathrm{s} (m_\mathrm{Z})$ is determined together with the PDFs. This approach is denoted as ‘PDF+$\alpha _\mathrm{s}$-fit’ in the following and uses inclusive DIS data [61–66] in addition to normalised jet cross section data [15, 21, 24], both measured by the H1 experiment [67–70].

## Cross section measurements

For the present analysis, measurements of jet cross sections and inclusive DIS cross sections in lepton-proton collisions performed by the H1 experiment at HERA are exploited.

*Jet cross sections* Cross sections for jet production in lepton-proton collisions have been measured by H1 at two different centre-of-mass energies using data from different periods of data taking. In the present analysis, inclusive jet and dijet cross sections measured in the range of negative four-momentum transfer squared $5<Q^{2}<15\,000\,\mathrm {GeV}^2$ and inelasticities $0.2<y<0.7$ are considered. An overview of the individual measurements [15, 17, 21, 23, 24] is given in Table 1. Common to all data, jets are defined in the Breit frame [3] using the $k_t$ clustering algorithm [71] with a resolution parameter $R=1$. The jet four-vectors are restricted to the pseudorapidity range $-1<\eta _\mathrm{lab}^\mathrm{jet}<2.5$ in the laboratory frame. The data sets ‘$300\,\mathrm {GeV}$’, ‘HERA-I’ and ‘HERA-II’ correspond to different data taking periods and are subdivided into two kinematic ranges, the low-$Q^{2}$ ($Q^{2}\lesssim 100\,\mathrm {GeV}^2$) and high-$Q^{2}$ ($Q^{2}\gtrsim 150\,\mathrm {GeV}^2$) domains, where different components of the H1 detector were used for the measurement of the scattered lepton.

**Table 1:** Summary of the kinematic ranges of the studied inclusive jet and dijet data sets. The *ep* centre-of-mass energy $\sqrt{s}$ and the integrated luminosity $\mathcal {L}$ are shown. Kinematic restrictions are made on the negative four-momentum transfer squared $Q^{2}$, the inelasticity *y* and the jet transverse momenta $P_\mathrm{T}^\mathrm{jet}$ as indicated. Common to all data sets is a requirement on the pseudorapidity of the jets, $-1<\eta _\mathrm{lab}^\mathrm{jet}<2.5$, not shown in the table. Dijet events are defined by extra cuts or on the average jet transverse momentum $\langle P_\mathrm{T} \rangle$ or the invariant mass of the two leading jets $m_{12}$. The asterisk denotes a cut not present in the original work [23] but imposed for the present analysis

Data set [ref.]	$\sqrt{s}$ $[\mathrm {GeV} ]$	$\mathcal {L}$ $[\mathrm{pb}^{-1}]$	DIS kinematic range	Inclusive jets	Dijets $n_\mathrm{jets}\ge 2$
$300\,\mathrm {GeV}$ [17]	300	33	$150<Q^{2}<5000\,\mathrm {GeV}^2$	$7<P_\mathrm{T}^\mathrm{jet}<50\,\mathrm {GeV}$	$P_\mathrm{T}^\mathrm{jet}>7\,\mathrm {GeV}$
			$0.2<y<0.6$		$8.5<\langle P_\mathrm{T} \rangle <35\,\mathrm {GeV}$
HERA-I [23]	319	43.5	$5<Q^{2}<100\,\mathrm {GeV}^2$	$5<P_\mathrm{T}^\mathrm{jet}<80\,\mathrm {GeV}$	$5<P_\mathrm{T}^\mathrm{jet}<50\,\mathrm {GeV}$
			$0.2<y<0.7$		$5<\langle P_\mathrm{T} \rangle <80\,\mathrm {GeV}$
					$$m_{12}>18\,\mathrm {GeV} $$
					$$(\langle P_\mathrm{T} \rangle >7\,\mathrm {GeV} )^*$$
HERA-I [21]	319	65.4	$150<Q^{2}<15000\,\mathrm {GeV}^2$	$5<P_\mathrm{T}^\mathrm{jet}<50\,\mathrm {GeV}$	−
			$$0.2<y<0.7$$		
HERA-II [15]	319	290	$5.5<Q^{2}<80\,\mathrm {GeV}^2$	$4.5<P_\mathrm{T}^\mathrm{jet}<50\,\mathrm {GeV}$	$P_\mathrm{T}^\mathrm{jet}>4\,\mathrm {GeV}$
			$0.2<y<0.6$		$5<\langle P_\mathrm{T} \rangle <50\,\mathrm {GeV}$
HERA-II [15, 24]	319	351	$150<Q^{2}<15000\,\mathrm {GeV}^2$	$5<P_\mathrm{T}^\mathrm{jet}<50\,\mathrm {GeV}$	$5<P_\mathrm{T}^\mathrm{jet}<50\,\mathrm {GeV}$
			$0.2<y<0.7$		$7<\langle P_\mathrm{T} \rangle <50\,\mathrm {GeV}$
					$$m_{12}>16\,\mathrm {GeV} $$

**Table 2:** H1 jet cross section measurements. Normalised dijet cross sections and statistical correlations between inclusive and dijet measurements are available only for the most recent measurements [15, 24]

Data set [ref.]	$Q^{2}$ domain	Inclusive jets	Dijets	Normalised inclusive jets	Normalised dijets	Stat. corr. between samples
$300\,\mathrm {GeV}$ [17]	High-$Q^{2}$	$\checkmark$	$\checkmark$	–	–	–
HERA-I [23]	Low-$Q^{2}$	$\checkmark$	$\checkmark$	–	–	–
HERA-I [21]	High-$Q^{2}$	$\checkmark$	–	$\checkmark$	–	–
HERA-II [15]	Low-$Q^{2}$	$\checkmark$	$\checkmark$	$\checkmark$	$\checkmark$	$\checkmark$
HERA-II [15, 24]	High-$Q^{2}$	$\checkmark$	$\checkmark$	$\checkmark$	$\checkmark$	$\checkmark$

The inclusive jet cross sections are measured double-differentially as functions of $Q^{2}$ and the jet transverse momentum in the Breit frame, $P_\mathrm{T}^\mathrm{jet}$, where the phase space is constrained by $Q^{2}$, *y*, $\eta _\mathrm{lab}^\mathrm{jet}$ and $P_\mathrm{T}^\mathrm{jet}$, as specified in Table 1.

For dijets at least two jets must be identified in the $\eta _\mathrm{lab}^\mathrm{jet}$ range above the relevant $P_\mathrm{T}^\mathrm{jet}$ threshold. The double-differential dijet cross sections are measured as functions of $Q^{2}$ and the average transverse momentum of the two leading jets, $\langle P_\mathrm{T} \rangle =(P_\mathrm{T}^\mathrm{jet1} + P_\mathrm{T}^\mathrm{jet2})/2$. In order to avoid regions of phase space where the predictions exhibit an enhanced infrared sensitivity [72, 73], the phase space definitions impose asymmetric cuts on the transverse momenta of the two leading jets [12]. Such an asymmetric cut may also be obtained by choosing $\langle P_\mathrm{T} \rangle$ larger than the minimum $P_\mathrm{T}^\mathrm{jet}$. For this reason, data points with $\langle P_\mathrm{T} \rangle < 7\,\mathrm {GeV}$ are excluded from the HERA-I low-$Q^{2}$ data set (Table 1).

**Table 3:** Summary of the inclusive NC and CC DIS data sets. The lepton type, the *ep* centre-of-mass energy $\sqrt{s}$ and the considered $Q^{2}$ range are shown. The numbers in parenthesis show the whole kinematic range of the data prior to applying the $Q^2$ cut specific for this analysis. The check-marks indicate the available measurements. The last column indicates cross sections determined with longitudinally polarised leptons

Data set [ref.]	Lepton type	$\sqrt{s}$ $[\mathrm {GeV} ]$	$Q^{2}$ range $[\mathrm {GeV}^2 ]$	NC cross sections	CC cross sections	Lepton beam polarisation
Combined low-$Q^{2}$ [64]	$e^+$	301,319	(0.5) 12–150	$\checkmark$	–	–
Combined low-$E_p$ [64]	$e^+$	225,252	(1.5) 12–90	$\checkmark$	–	–
94–97 [61]	$e^+$	301	150–30 000	$\checkmark$	$\checkmark$	–
98–99 [62, 63]	$e^-$	319	150–30 000	$\checkmark$	$\checkmark$	–
99–00 [63]	$e^+$	319	150–30 000	$\checkmark$	$\checkmark$	–
HERA-II [65]	$e^+$	319	120–30 000	$\checkmark$	$\checkmark$	$\checkmark$
HERA-II [65]	$e^-$	319	120–50 000	$\checkmark$	$\checkmark$	$\checkmark$

Data from different periods and $Q^{2}$ ranges are statistically independent, whereas dijet and inclusive jet data of the same data set are statistically correlated. These correlations have been determined for the HERA-II data sets [15, 24]. Different data sets, as well as inclusive jet and dijet data of the same data set, may furthermore share individual sources of experimental uncertainties [15, 56] and thus correlations are present for all data points considered.

*Normalised jet cross sections* The more recent data sets [15, 21, 24] also include measurements where the jet cross sections are normalised to the inclusive NC DIS cross section of the respective $Q^{2}$ interval, as indicated in Table 2. Correlations of systematic and statistical uncertainties partially cancel for the ratio of jet cross sections and inclusive NC DIS cross sections. Therefore, normalised jet cross sections are ideally suited for studies together with inclusive NC DIS data.

*Inclusive DIS cross sections* In order to constrain the parameters of the PDFs in the PDF+$\alpha _\mathrm{s}$-fit, polarised and unpolarised inclusive NC and CC (charged current) DIS cross sections [61–66] measured by the H1 experiment are used in addition. Data taken during different data taking periods and with different centre-of-mass energies are considered and a summary of these measurements is given in Table 3. This data sample is identical to the one used in the H1PDF2012 PDF fit [65], where correlations of experimental uncertainties have been quantified. Inclusive DIS and jet cross sections are statistically and experimentally correlated. These correlations are taken into account by using normalised jet cross sections.

## Determination of ${\varvec{\alpha _\mathrm{s} (m_\mathrm{Z})}}$ from H1 jet cross sections

The strong coupling constant $\alpha _\mathrm{s} (m_\mathrm{Z})$ is determined from inclusive jet and dijet cross sections in NC DIS measured by the H1 collaboration and using NNLO QCD predictions.

### Predictions

The cross sections for inclusive jet and dijet production for a given phase space interval *i* (for instance a ‘bin’ in the relevant physical observables) are calculated [4, 74] as a convolution in the variable *x* of the PDFs $f_k$ and perturbatively calculated partonic cross sections ${\hat{\sigma }}_{i,k}$,

1 $$\begin{aligned} \sigma _i = \sum _{k=g,q,\overline{q}} \int dx f_{k} (x,\mu _\mathrm{F}) \hat{\sigma }_{i,k}(x,\mu _\mathrm{R},\mu _\mathrm{F}) \cdot c_{\mathrm{had},i}, \end{aligned}$$

where the sum runs over all parton flavours *k*. The calculations depend on the renormalisation scale $\mu _\mathrm{R}$ and the factorisation scale $\mu _\mathrm{F}$. The factors $c_{\mathrm{had},i}$ account for non-perturbative effects (hadronisation corrections).

**Fig. 17:**
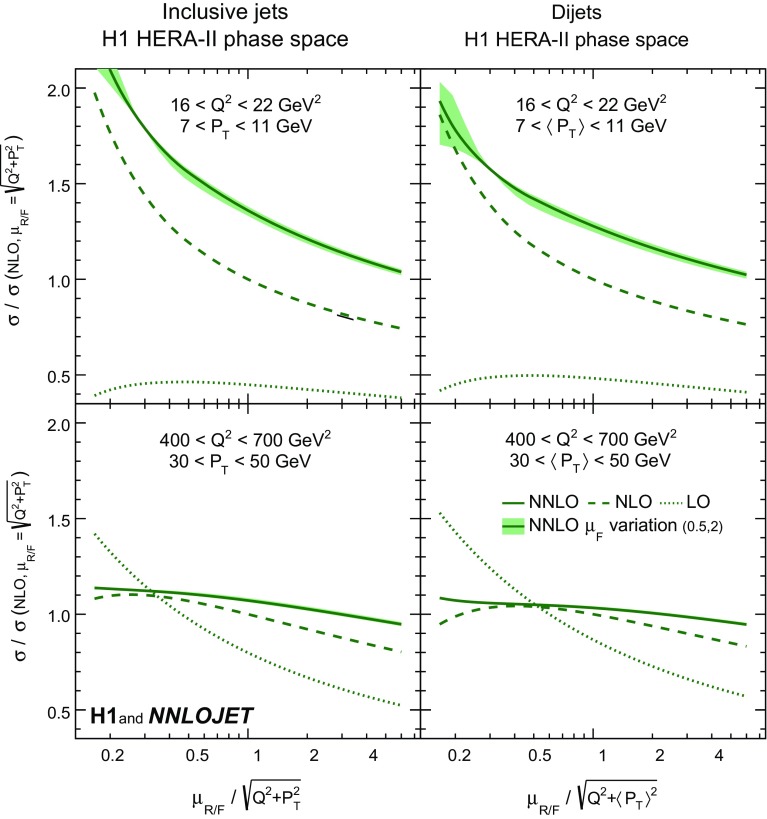
Relative change of jet cross section as a function of a multiplicative factor applied to the renormalisation and factorisation scale for four exemplary data points of the HERA-II phase space. The bin definitions are displayed in the respective panels. The left panels show inclusive jet cross sections, and the right panels dijet cross sections. The full line shows the cross section dependence for the NNLO, the dashed line for NLO and the dotted line for LO calculations. For better comparison, all calculations are performed with the same PDF set (NNPDF3.1 NNLO). For all panels, the cross sections are normalised to the respective NLO cross section with unity scale factor. The filled area around the NNLO calculation indicates variations of the factorisation scale by factors of 0.5 and 2 around the chosen value for $\mu _\mathrm{R}$

Both the $f_k$ and the ${\hat{\sigma }}_{i,k}$ are sensitive to the strong coupling. The partonic cross sections are given in terms of the perturbative expansion in orders of $\alpha _\mathrm{s}(\mu _\mathrm{R})$

2 $$\begin{aligned} \hat{\sigma }_{i,k} = \sum _{n} \alpha _\mathrm{s} ^{n}(\mu _\mathrm{R})\hat{\sigma }_{i,k}^{(n)}(x,\mu _\mathrm{R},\mu _\mathrm{F}). \end{aligned}$$

For high $P_{T}$ jet production in the Breit frame the lowest order is $n=1$. The hard coefficients $\hat{\sigma }_{i,k}^{(n)}$ are calculated for the expansion up to $\mathcal {O} (\alpha _\mathrm{s} ^3)$ taking into account properties of the jet algorithm in the integration over the phase space. The renormalisation scale dependence (‘running’) of the coupling satisfies the renormalisation group equation

3 $$\begin{aligned} \mu _\mathrm{R} ^2\frac{d\alpha _\mathrm{s}}{d\mu _\mathrm{R} ^2} = \beta (\alpha _\mathrm{s}). \end{aligned}$$

The QCD beta-function $\beta$ is known at 4-loop accuracy [75, 76]. The strength of the coupling thus may be determined at an arbitrary scale, which is conventionally chosen to be the mass of the *Z*-boson, $m_\mathrm{Z} =91.1876\,\mathrm {GeV}$ [2]. Here, the calculations are performed in the modified minimal subtraction ($\overline{\mathrm{MS}}$) scheme in 3-loop accuracy and using 5 flavors, $\alpha _\mathrm{s}(\mu _\mathrm{R}) =\alpha ^{(5)}_{\overline{\mathrm{MS}}}(\mu _\mathrm{R})$.

The PDFs $f_k$ exhibit a dependence on $\alpha _\mathrm{s} (m_\mathrm{Z})$, which originates from the factorisation theorem [74]. This dependence can be schematically expressed as [77–79]

4 $$\begin{aligned} \mu _\mathrm{F} ^2\frac{d f}{d\mu _\mathrm{F} ^2} = \mathcal {P}(\alpha _\mathrm{s})\otimes f \end{aligned}$$

with $\mathcal {P}$ being the QCD splitting kernels and the symbol ‘$\otimes$’ denoting a convolution. After fixing the *x*-dependence of the PDFs $f_k$ at a scale $\mu _{0}$ and setting $\mu _\mathrm{R} =\mu _\mathrm{F}$, the PDF at any factorisation scale $\mu _\mathrm{F}$ is calculated as

5 $$\begin{aligned} f\left( x,\mu _\mathrm{F},\alpha _\mathrm{s} (m_\mathrm{Z}) \right) = \Gamma \left( \mu _\mathrm{F},\mu _{0},\alpha _\mathrm{s} (m_\mathrm{Z}) \right) \otimes f_{\mu _{0}}\left( x\right) \end{aligned}$$

with $\Gamma$ being the evolution kernel which obeys Eq. (4). It is here calculated in NNLO, i.e. in 3-loop accuracy [80, 81], with five active flavours.

The evolution starting scale is chosen to be $\mu _{0} =20\,\mathrm {GeV}$. This is a typical scale of the jet data studied. As a consequence, the influence of the evolution of Eq. (5) on the $\alpha _\mathrm{s}$ determination is moderate, because $\mu _\mathrm{F} \approx \mu _{0}$. The PDFs at that scale are well known, in particular the quark densities. Moreover, the latter are to a large extent insensitive to the assumption made on the strong coupling $\alpha _\mathrm{s} ^\mathrm{PDF}(m_\mathrm{Z})$ during their determination, because in leading order QCD inclusive DIS is independent of $\alpha _\mathrm{s}$ Ṫhe gluon density is constrained due to QCD sum-rules and the precisely known quark densities. In the vicinity of a scale of $20\,\mathrm {GeV}$ threshold effects from heavy quarks are not relevant. The PDFs at $\mu _{0} =20\,\mathrm {GeV}$ are provided by the NNPDF3.1 PDF set [82] which was obtained with a nominal value of $\alpha _\mathrm{s} ^\mathrm{PDF}(m_\mathrm{Z}) =0.118$. The influence of those choices is quantified in Sect. 3.3.

The scales $\mu _\mathrm{R}$ and $\mu _\mathrm{F}$ are chosen to be

6 $$\begin{aligned} \mu _\mathrm{R} ^2=\mu _\mathrm{F} ^2=Q^{2}+P_{T}^2, \end{aligned}$$

where $P_{T}$ denotes $P_\mathrm{T}^\mathrm{jet}$ in the case of inclusive jet cross sections and $\langle P_\mathrm{T} \rangle$ for dijets. Previously, a variety of different scale definitions have been employed by H1 [15, 17, 21, 23, 24, 33–35, 83], ZEUS [26, 27, 29–32, 55] and elsewhere [18, 72, 84–89]. The choice adopted here was already suggested and discussed earlier [25, 28, 90, 91]. Advantages of the scale defined in Eq. (6) are in its simple functional form and in the fact that it remains non-zero in either of the kinematical limits $Q^{2}\rightarrow 0\,\mathrm {GeV}^2$ and $P_{T}^2\ll Q^{2}$. This is particularly important here, since low- and high-$Q^{2}$ domains and a large range in $P_{T}$ are considered.

The inclusive jet and dijet NNLO predictions as a function of $\mu _\mathrm{R}$ and $\mu _\mathrm{F}$ are studied for selected phase space regions in Fig. 1. The dependence on the scale factor is strongest for cross sections at lower values $\mu _\mathrm{R}$, i.e. lower values of $Q^{2}$ and $P_{T}$. The NNLO predictions depend less on the scale factor than the NLO predictions. Other choices of $\mu _\mathrm{R}$ and $\mu _\mathrm{F}$ are studied with the $\alpha _\mathrm{s}$ fit in Sect. 3.3.

The dependence of the inclusive jet and dijet NNLO predictions on $\alpha _\mathrm{s} (m_\mathrm{Z})$ is displayed in Fig. 2, where the two contributions to the $\alpha _\mathrm{s} (m_\mathrm{Z})$ dependence, $\hat{\sigma }_{ik}$ and $f_k$, are separated. The predominant sensitivity to $\alpha _\mathrm{s} (m_\mathrm{Z})$ arises from $\hat{\sigma }_{i,k}$.

**Fig. 18:**
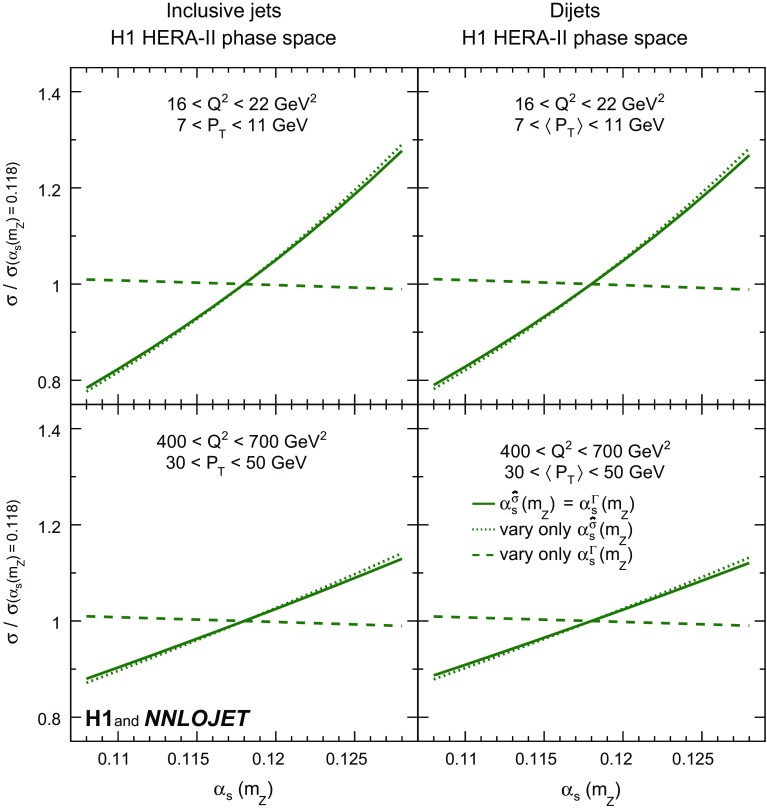
Relative change of jet cross section as a function of $\alpha _\mathrm{s} (m_\mathrm{Z})$ for four exemplary data points of the HERA-II phase space. The bin definitions are displayed in the respective panels. The left panels show inclusive jet cross sections, and the right pads dijet cross sections. The full line indicates the cross section dependence as a function of $\alpha _\mathrm{s} (m_\mathrm{Z})$, while the dotted line illustrates the dependence where $\alpha _\mathrm{s} (m_\mathrm{Z})$ is varied only in the partonic cross sections and the dashed line illustrates a variation only in the PDF evolution starting from $\mu _{0} =20\,\mathrm {GeV}$. The cross sections are normalised to the nominal cross section defined with $\alpha _\mathrm{s} (m_\mathrm{Z}) =0.118$

The hard coefficients $\hat{\sigma }^{(n)}_{i,k}$ are calculated using the program  [11, 12, 60], which is interfaced to fastNLO [92] to allow for computationally efficient, repeated calculations with different values of $\alpha _\mathrm{s} (m_\mathrm{Z})$, different scale choices and different PDF sets. The PDFs are included in the LHAPDF package [93]. The evolution kernels are calculated using the program APFEL++ [94] and all results are validated with the programs APFEL [95] and QCDNUM [96, 97]. The $\alpha _\mathrm{s}$ evolution is calculated using the APFEL++ code and validated with the CRunDec code [98], and the running of the electromagnetic coupling with $Q^{2}$ is calculated using the package EPRC [99, 100]. The fits are performed using the Alpos fitting framework [101].

### Methodology

The value of the strong coupling constant is determined in a fit of theory predictions to H1 jet cross sections with a single free fit parameter. The goodness-of-fit quantity, which is subject to the minimisation algorithm, is defined as

7 $$\begin{aligned} \chi ^{2}= & {} \sum _{i} \sum _{j} \left( \log \varsigma _i - \log \sigma _i\right) (V_\mathrm{exp} +V_\mathrm{had}\nonumber \\&+\,V_\mathrm{PDF})^{-1}_{ij}(\log \varsigma _j - \log \sigma _j), \end{aligned}$$

where $\varsigma _i$ are the measurements and $\sigma _i$ the predictions (Eq. (1)). The covariance matrices express the relative uncertainties of the data ($V_\mathrm{exp}$), hadronisation correction factors ($V_\mathrm{had}$) and the PDFs ($V_\mathrm{PDF}$). The underlying statistical model is that the logarithm of each measurement is normal-distributed within its relative uncertainty, or equivalently the measurements follow log-normal distributions. The fit value is found using the TMinuit algorithm [102, 103]. Correlations of the uncertainties among the different data sets and running periods are considered [15, 56]. The hadronisation corrections and their uncertainties have been provided together with the jet cross section measurements [15, 17, 21, 23, 24]. The PDF uncertainties were provided by the authors of the respective PDF set.

To each data point a representative scale value $\tilde{\mu }$ is assigned, which is calculated from the geometric mean of the bin boundaries (denoted as ‘dn’ and ‘up’) in $Q^{2}$ and $P_{T}$,

8 $$\begin{aligned} Q^2_{\mathrm{avg},i} = \sqrt{ Q^2_{\mathrm{dn},i} Q^2_{\mathrm{up},i}} \quad \mathrm{and}\quad P_{\mathrm{T,avg},i} = \sqrt{ P_{\mathrm{T,dn},i} P_{\mathrm{T,up},i} }, \end{aligned}$$

together with the definition of the scales in Eq. (6) as

9 $$\begin{aligned} \tilde{\mu }^2_i = Q^2_{\mathrm{avg},i} + P^2_{\mathrm{T,avg},i}. \end{aligned}$$

Effects from heavy quark masses become important at lower scales, while the NNLO calculations are performed with five massless quark flavours. Unless otherwise stated the data are selected with the condition $\tilde{\mu } >2m_b$, with $m_b=4.5\,\mathrm {GeV}$ [56] being the mass of the *b*-quark.

The uncertainty calculated by TMinuit contains the experimental (exp), hadronisation (had) and PDF uncertainties (PDF). The breakdown of the uncertainties into these three components is obtained from repeated fits with $V_\mathrm{had}$ and/or $V_\mathrm{PDF}$ set to zero. Further uncertainties are defined in Sect. 3.3 and will be denoted as PDFset, PDF$\alpha _\mathrm{s}$, and scale uncertainties. The theory uncertainty (‘th’) is defined as the quadratic sum of the PDF, PDFset, PDF$\alpha _\mathrm{s}$, hadronisation and scale uncertainties, and the ‘total’ uncertainty considers additionally the experimental uncertainty.

The value of $\alpha _\mathrm{s} (m_\mathrm{Z})$ is determined separately for each individual data set, for all inclusive jet measurements, for all dijet measurements, and for all H1 jet data taken together. The latter is denoted as ‘H1 jets’ in the following. In the case of fits to ‘H1 jets’, dijet data from the HERA-I running period however are excluded, since their statistical correlations to the respective inclusive jet data are not known (Table 2).

### Sensitivity of the fit to input parameters

*Sensitivity to* ${{\alpha _\mathrm{s} (m_\mathrm{Z})}}$ The sensitivity of the data to $\alpha _\mathrm{s} (m_\mathrm{Z})$ and the consistency of the calculations are investigated by performing fits with two free parameters representing the two distinct appearances of $\alpha _\mathrm{s} (m_\mathrm{Z})$ in Eq. (1), i.e. in the PDF evolution, $\alpha _\mathrm{s} ^{\Gamma }(m_\mathrm{Z})$, and in the partonic cross sections, $\alpha _\mathrm{s} ^{\hat{\sigma }}(m_Z)$. The cross sections with the $\alpha _\mathrm{s}$ contributions identified separately are schematically expressed by

10 $$\begin{aligned} \sigma _i = f \left( \alpha _\mathrm{s} ^{\Gamma }(m_\mathrm{Z}) \right) \otimes \hat{\sigma }_{i}\left( \alpha _\mathrm{s} ^{\hat{\sigma }}(m_Z)\right) \cdot c_{\mathrm{had},i}, \end{aligned}$$

where $\alpha _\mathrm{s} (m_\mathrm{Z})$ as of Eq. (5) is denoted as $\alpha _\mathrm{s} ^{\Gamma }(m_\mathrm{Z})$, and $\alpha _\mathrm{s} (m_\mathrm{Z})$ as of Eq. (2) is denoted as $\alpha _\mathrm{s} ^{\hat{\sigma }}(m_Z)$. The result of such a fit performed for H1 jets is displayed in Fig. 3. Consistency is found for the two fitted values of $\alpha _\mathrm{s} (m_\mathrm{Z})$, where the resulting $\alpha _\mathrm{s} ^{\Gamma }(m_\mathrm{Z})$ tends to be larger than $\alpha _\mathrm{s} ^{\hat{\sigma }}(m_Z)$. It is observed that the predominant sensitivity to $\alpha _\mathrm{s} (m_\mathrm{Z})$ arises from the $\hat{\sigma }_{i,k}$, as was already suggested by the jet cross section study (Fig. 2). The ellipses obtained using PDFs determined with values $\alpha _\mathrm{s} ^\mathrm{PDF}(m_\mathrm{Z})$ of 0.116, 0.118 and 0.120 are consistent with each other. In the following, all fits are performed using a single fit parameter $\alpha _\mathrm{s} (m_\mathrm{Z})$.

**Fig. 19:**
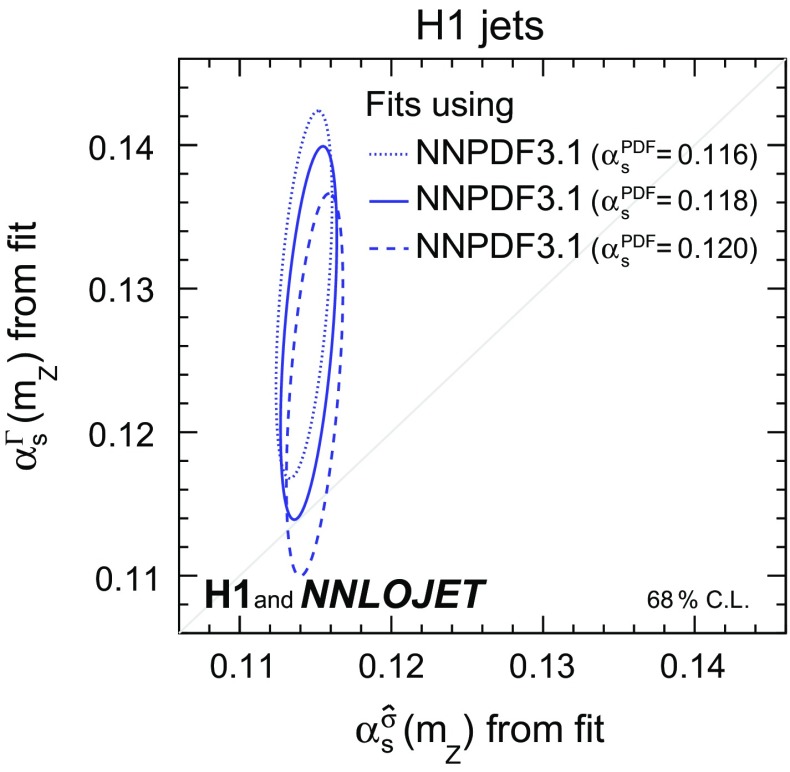
Results from fits to H1 jets with two free fit parameters for $\alpha _\mathrm{s} (m_\mathrm{Z})$, where the appearances of $\alpha _\mathrm{s} (m_\mathrm{Z})$ in the PDF evolution $\alpha _\mathrm{s} ^{\Gamma }(m_\mathrm{Z})$ and in the partonic cross sections $\alpha _\mathrm{s} ^\sigma (m_\mathrm{Z})$ are identified separately. The ellipses display a confidence level of $68\,\%$ including the experimental, hadronisation and PDF uncertainties, and thus the lines are calculated for $\Delta \chi ^{2}=2.3$. The dotted, full and dashed lines indicate the contour for $\Delta \chi ^{2}=2.3$ using three versions of the NNPDF3.1 set which were obtained using values for $\alpha _\mathrm{s} ^\mathrm{PDF}(m_\mathrm{Z})$ of 0.116, 0.118 and 0.120, respectively

*Dependence on the choice of PDF* Values of $\alpha _\mathrm{s} (m_\mathrm{Z})$ are determined for other PDF sets and for alternative values $\alpha _\mathrm{s} ^\mathrm{PDF}(m_\mathrm{Z})$.

The results obtained using different PDFs are displayed in Fig. 4 for fits to inclusive jet and dijet cross sections, and in Fig. 5 for H1 jets. In Fig. 5 (right) only H1 jets with $\tilde{\mu } >28\,\mathrm {GeV}$ are used. The predictions using NNPDF3.1, determined with $\alpha _\mathrm{s} ^\mathrm{PDF}(m_\mathrm{Z}) =0.118$, provide good description of the data with $\chi ^{2}$/$n_\mathrm{dof}$ close to unity (Fig. 4), where $n_\mathrm{dof}$ denotes the number of data points minus one. The fitted $\alpha _\mathrm{s} (m_\mathrm{Z})$ values are only weakly correlated to the $\alpha _\mathrm{s} ^\mathrm{PDF}(m_\mathrm{Z})$ values employed for the PDF extraction (Figs. 4 and 5). Different PDF sets yield consistent results. The correlation of $\alpha _\mathrm{s} ^\mathrm{PDF}(m_\mathrm{Z})$ and the fitted $\alpha _\mathrm{s} (m_\mathrm{Z})$ vanishes when using only data with $\tilde{\mu } >28\,\hbox {GeV}$.

**Fig. 20:**
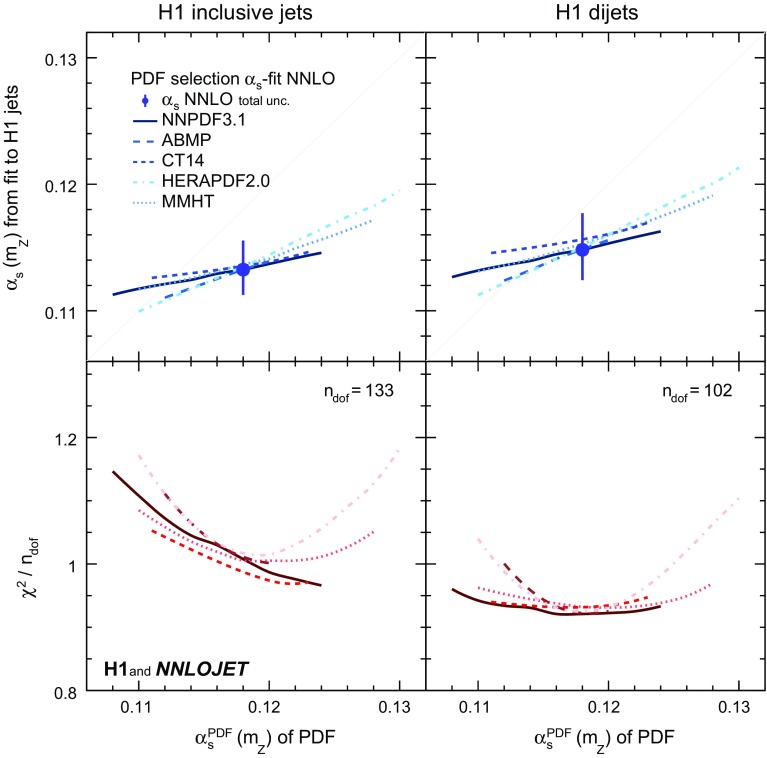
Dependencies of the fitted values of $\alpha _\mathrm{s} (m_\mathrm{Z})$ on the input PDFs for separate fits of inclusive jet and dijet data. Shown are fits using the ABMP, CT14, HERAPDF2.0, MMHT and NNPDF3.1 PDF sets. For each case, the PDFs are available for different input values $\alpha _\mathrm{s} ^\mathrm{PDF}(m_\mathrm{Z})$ used for the PDF determination, and these values are displayed on the horizontal axis. The PDFs are available only for discrete values of $\alpha _\mathrm{s} ^\mathrm{PDF}(m_\mathrm{Z})$ and the results are connected by smooth lines. The lower panel displays the resulting values of $\chi ^{2}/n_\mathrm{dof}$ of the fits

**Fig. 21:**
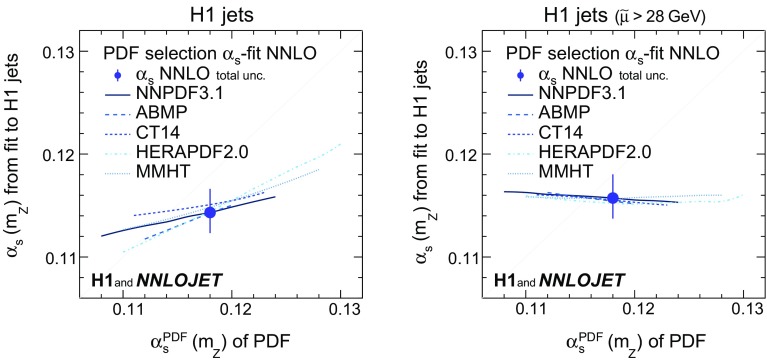
Dependencies of the fitted values of $\alpha _\mathrm{s} (m_\mathrm{Z})$ on the input PDFs for the H1 jets fit (left) and the H1 jets fit with $\tilde{\mu } >28\,\mathrm {GeV}$ (right). Further details are given in the caption of Fig. 4

Three PDF related uncertainties are assigned to the fitted $\alpha _\mathrm{s} (m_\mathrm{Z})$ results. The ‘PDF’ uncertainty originates from the data used for the PDF extraction [82]. A ‘PDFset’ uncertainty is defined as half of the maximum difference of the results from fits using the ABMP [104], CT14 [105], HERAPDF2.0 [56], MMHT [58] or NNPDF3.1 PDF set [82]. The ‘PDF$\alpha _\mathrm{s}$ ’ uncertainty is defined as the difference of results from repeated fits using PDFs of the NNPDF3.1 series determined with $\alpha _\mathrm{s} ^\mathrm{PDF}(m_\mathrm{Z})$ values differing by 0.002 [106]. This uncertainty can be considered to be uncorrelated to the PDF uncertainty [106, 107]. The size of the variation includes the NNPDF3.1 PDF set determined with $\alpha _\mathrm{s} ^\mathrm{PDF}(m_\mathrm{Z}) =0.116$, where $\alpha _\mathrm{s} ^\mathrm{PDF}(m_\mathrm{Z})$ is close to the fitted $\alpha _\mathrm{s} (m_\mathrm{Z})$, in particular when restricting H1 jets to $\tilde{\mu } >28\,\mathrm {GeV}$ (Fig. 5). The variation of $\mu _{0}$ in the range 10 to 90 GeV is also studied but has negligible effect on the results.

*Scale variants and comparison of NLO and NNLO predictions* Studies of different choices for $\mu _\mathrm{R}$ and $\mu _\mathrm{F}$ are commonly used to estimate contributions of higher orders beyond NNLO.

The dependence of the results on $\mu _\mathrm{R}$ and $\mu _\mathrm{F}$ is studied by applying scale factors to the definition of $\mu _\mathrm{R}$ and $\mu _\mathrm{F}$. The values of $\alpha _\mathrm{s} (m_\mathrm{Z})$ and $\chi ^{2}/n_\mathrm{dof}$ resulting from the fits to inclusive jet and to dijet cross sections are displayed in Fig. 6 indicating that the standard choice for the scales (unity scale factor) yields reasonable values of $\chi ^{2}/n_\mathrm{dof}$. Figure 7 displays the resulting $\alpha _\mathrm{s} (m_\mathrm{Z})$ for fits to H1 jets. In general, variations of $\mu _\mathrm{R}$ have a larger impact on the result than those of $\mu _\mathrm{F}$. When restricting the data to $\tilde{\mu } >28\,\mathrm {GeV}$, the scale dependence is greatly reduced.

**Fig. 22:**
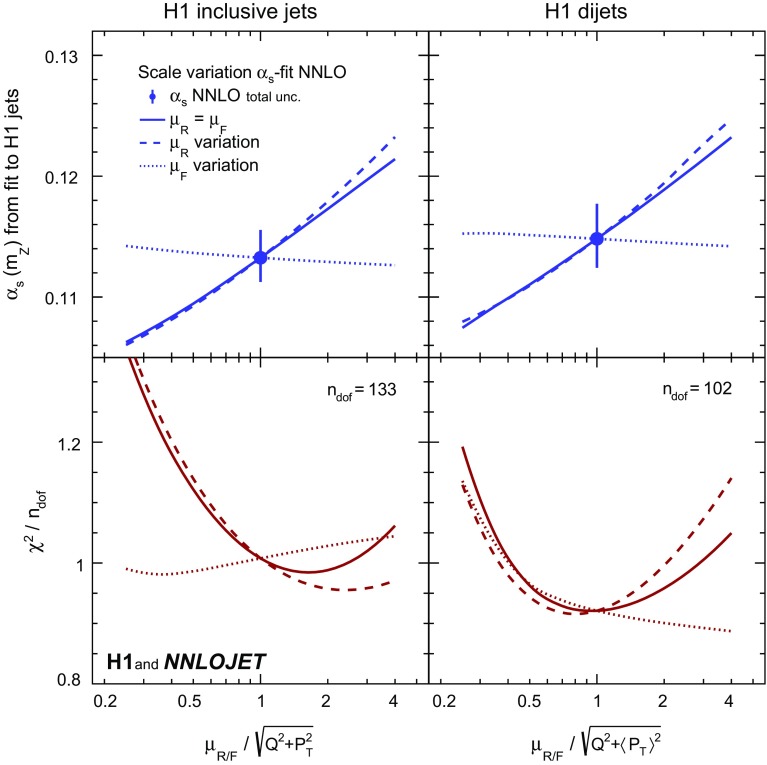
Dependencies of the fitted values of $\alpha _\mathrm{s} (m_\mathrm{Z})$ as a function of the scale factors applied to the renormalisation and factorisation scales ($\mu _\mathrm{R}$ and $\mu _\mathrm{F}$) for separate fits of inclusive jet and dijet data. The upper panels show the fitted value of $\alpha _\mathrm{s} (m_\mathrm{Z})$, and the lower panels show the values of $\chi ^{2}/n_\mathrm{dof}$. The left (right) panels show the values for the fit to inclusive jet (dijet) cross sections. The solid lines show the effects from varying $\mu _\mathrm{R}$ and $\mu _\mathrm{F}$ together. The dashed (dotted) lines show the effects from varying $\mu _\mathrm{R}$ ($\mu _\mathrm{F}$) alone

**Fig. 23:**
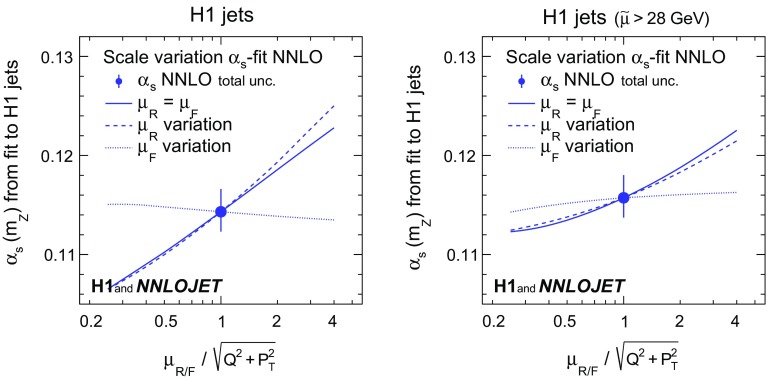
Dependencies of the fitted values of $\alpha _\mathrm{s} (m_\mathrm{Z})$ as a function of the scale factors for the H1 jets fit (left) and the H1 jets fit with $\tilde{\mu } >28\,\mathrm {GeV}$ (right). Further details are given in the caption of Fig. 6

Scale uncertainties are estimated through repeated fits with scale factors applied simultaneously to $\mu _\mathrm{R}$ and $\mu _\mathrm{F}$. Instead of varying the scales up and down by conventional factors, in this analysis a linear error propagation to the scale factors of 0.5 and 2 is performed using the derivative determined at the nominal scale. This is justified by the almost linear dependence on the logarithm of the scale factor (Figs. 6 and 7) and thus symmetric scale uncertainties are presented.

Alternative choices for $\mu _\mathrm{R}$ and $\mu _\mathrm{F}$ are investigated and the results for $\alpha _\mathrm{s} (m_\mathrm{Z})$ with values of $\chi ^{2}$/$n_\mathrm{dof}$ are displayed in Fig. 8 for fits to inclusive jet and dijet data. The nominal scale definition $\mu _\mathrm{R} ^2=\mu _\mathrm{F} ^2=Q^{2}+P_{T}^2$ results in good agreement of theory and data in terms of $\chi ^{2}$/$n_\mathrm{dof}$. The results obtained with alternative scale choices typically vary within the assigned scale uncertainty. This is also observed for fits to H1 jets, presented in Fig. 9. A representative scale of the jet data analysed here is $20\,\mathrm {GeV}$. Using $\mu _\mathrm{R} =\mu _\mathrm{F} =\mu _{0} =20\,\mathrm {GeV}$, simplifies the theory calculations such that Eqs. (3)–(5) are not used and no running of the coupling or evolution of the PDFs is needed. For this scale choice the resulting value of $\alpha _\mathrm{s} (m_\mathrm{Z})$ (which after the fit is evolved from $20\,\mathrm {GeV}$ to $m_\mathrm{Z}$ for comparisons) is also found to be consistent with the values obtained using the nominal scale.

**Fig. 24:**
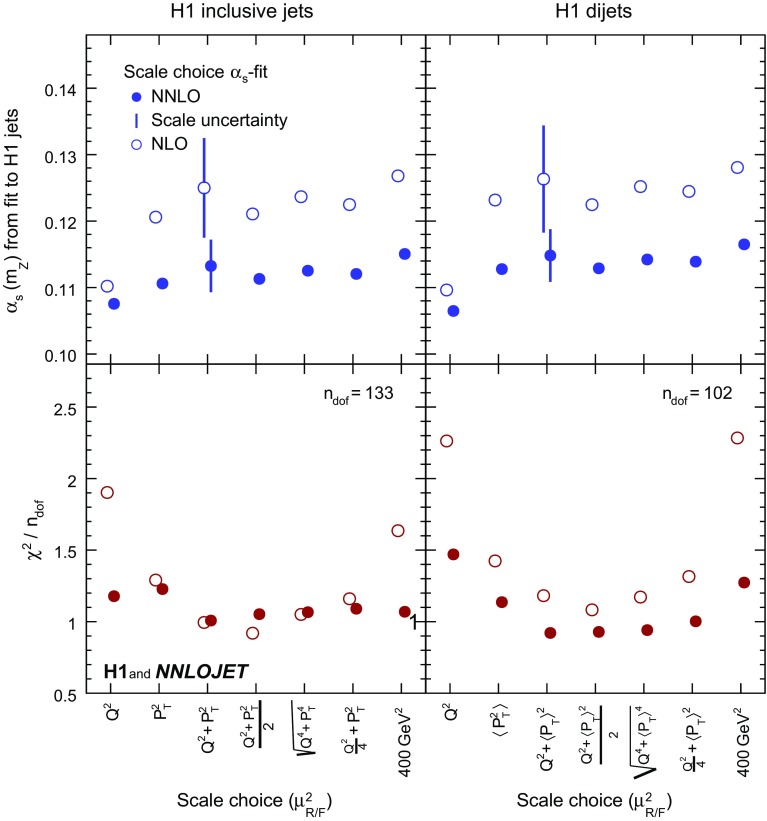
Values of $\alpha _\mathrm{s} (m_\mathrm{Z})$ obtained for various different definitions of the renormalisation and factorisation scales ($\mu _\mathrm{R}$ and $\mu _\mathrm{F}$) in separate fits of inclusive jet and dijet data. The lower panels show $\chi ^{2}$/$n_\mathrm{dof}$ of the fits. The open circles display results obtained using NLO matrix elements. The vertical bars indicate the scale uncertainties displayed together with the nominal scale choice

**Fig. 25:**
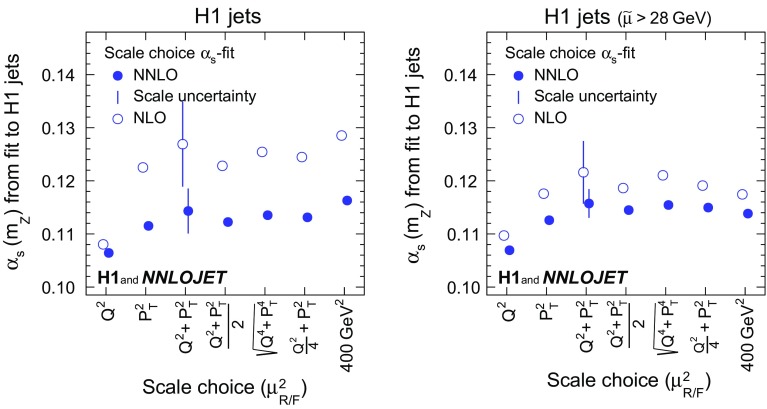
Values of $\alpha _\mathrm{s} (m_\mathrm{Z})$ obtained for various different definitions of the renormalisation and factorisation scales ($\mu _\mathrm{R}$ and $\mu _\mathrm{F}$) in the H1 jets fit (left) and the H1 jets fit with $\tilde{\mu } >28\,\mathrm {GeV}$ (right). The open circles display results obtained using NLO matrix elements. The vertical bars indicate the scale uncertainties displayed together with the nominal scale choice

The fits are repeated with the partonic cross sections $\hat{\sigma }_{i,k}$ calculated only up to NLO where for better comparisons identical scale definitions and identical PDFs determined in NNLO fits are used. For inclusive jets, the values of $\chi ^{2}$/$n_\mathrm{dof}$ of the NLO fits are of comparable size for some of the studied scale choices, but are significantly worse for certain choices such as $\mu _\mathrm{R} ^2=\mu _\mathrm{F} ^2=Q^{2}$. For dijets, the values of $\chi ^{2}$/$n_\mathrm{dof}$ are always higher for NLO than for NNLO calculations. The NLO calculations exhibit an enhanced sensitivity to the choice of the scale and to scale variations, as compared to NNLO, resulting in scale uncertainties of $\alpha _\mathrm{s} (m_\mathrm{Z})$ of 0.0077, 0.0081 and 0.0083 for inclusive jets, dijet and H1 jets, respectively, as compared to uncertainties of 0.0040, 0.0040 and 0.0042 in NNLO, respectively. The previously observed reduction of scale uncertainties of the cross section predictions at NNLO [11, 12, 15] is reflected in a corresponding reduction of the $\alpha _\mathrm{s} (m_\mathrm{Z})$ scale uncertainties.

*Restricting the scale* ${{\tilde{\mu }}}$ In order to study the size of the uncertainties as a function of $\tilde{\mu }$, the fits to inclusive jet and to dijet cross sections are repeated using data points exceeding a given value $\tilde{\mu } _\mathrm{cut}$. The resulting uncertainties are displayed in Fig. 10. The experimental uncertainties are smaller for lower $\tilde{\mu }$. This is because more data are considered in the fit, but also since the data at lower values of $\tilde{\mu }$ have an enhanced sensitivity to $\alpha _\mathrm{s} (m_\mathrm{Z})$ due to the running of the strong coupling. In contrast, the scale uncertainties of the NNLO cross section predictions are largest for low values of $\tilde{\mu }$, and thus decrease with increasing $\tilde{\mu }$. Considering only data with values of $\tilde{\mu }$ above approximately $30\,\mathrm {GeV}$ the experimental and scale uncertainty become similar in size.

**Fig. 26:**
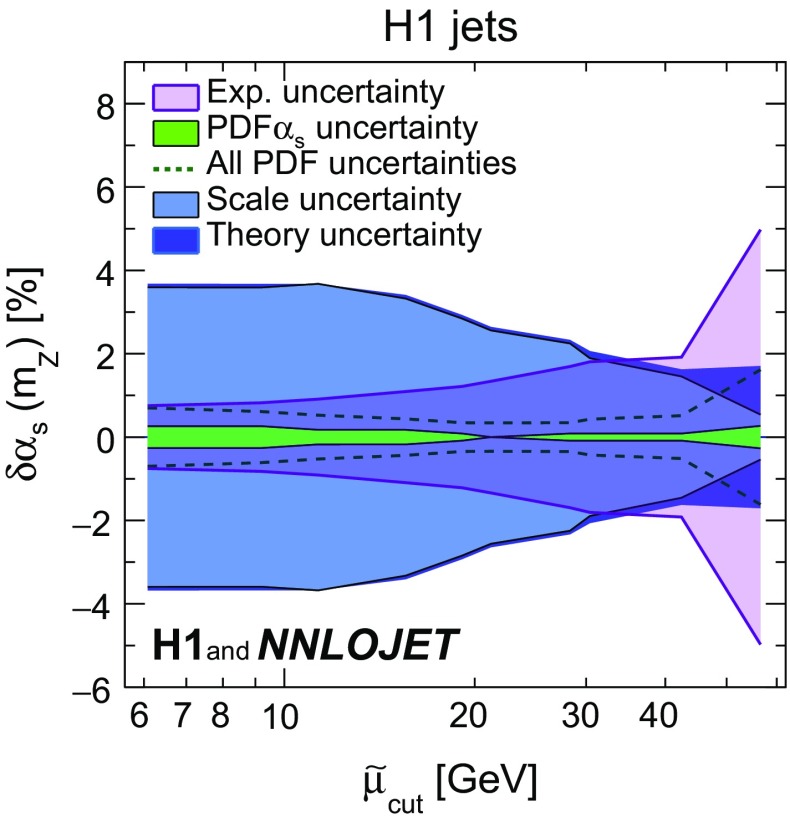
Uncertainties of the $\alpha _\mathrm{s}$ fit as a function of the parameter $\tilde{\mu } _\mathrm{cut}$ which restricts the jet data to high scales. The experimental, scale, PDF$\alpha _\mathrm{s}$, quadratic sum of all PDF related uncertainties, and the theory uncertainty are shown

The result obtained with $\tilde{\mu } >28\,\mathrm {GeV}$ is considered as the main result of this article.

At values of $\tilde{\mu } _\mathrm{cut}$ around $20\,\mathrm {GeV}$ the PDF$\alpha _\mathrm{s}$ uncertainty effectively vanishes. In other words, the fit result is insensitive to the $\alpha _\mathrm{s} ^\mathrm{PDF}(m_\mathrm{Z})$ assumptions made for the PDF determination. A possible explanation is the gradual change of the fraction of gluon and quark induced processes with $\tilde{\mu }$: data at lower values of $\tilde{\mu }$ have contributions from low-*x* where the gluon PDF is dominating, whereas data at higher values of $\tilde{\mu }$ have a successively higher fraction of quark induced processes. The quark PDFs are less dependent on $\alpha _\mathrm{s} ^\mathrm{PDF}(m_\mathrm{Z})$ than the gluon PDF, and are well determined by inclusive DIS data.

### Results

*The value of the strong coupling constant* ${{\alpha _\mathrm{s} (m_\mathrm{Z})}}$ The values of $\alpha _\mathrm{s} (m_\mathrm{Z})$ obtained from the fits to the data are collected in Table 4 and displayed in Fig. 11. Good agreement between theory and data is found.

**Table 4:** Summary of values of $\alpha _\mathrm{s} (m_\mathrm{Z})$ from fits to H1 jet cross section measurements using NNLO predictions. The uncertainties denote the experimental (exp), hadronisation (had), PDF, PDF$\alpha _\mathrm{s}$, PDFset and scale uncertainties as described in the text. The rightmost three columns denote the quadratic sum of the theoretical uncertainties (th), the total (tot) uncertainties and the value of $\chi ^{2}/n_\mathrm{dof}$ of the corresponding fit. Along the vertical direction, the table data are segmented into five parts. The uppermost part summarises fits to individual inclusive jet datasets. The second part corresponds to fits of the individual dijet datasets. The third part summarises fits to all inclusive jets or all dijets together, with different choices of the lower cut on the scale $\tilde{\mu } _{\mathrm{cut}}$. The fourth group of fits, labelled H1 jets, is made using all available dijet and inclusive jet data together, for three different choices of $\tilde{\mu } _{\mathrm{cut}}$. The bottom row corresponds to a combined fit of inclusive data and normalised jet data. For that fit, theoretical uncertainties related to the PDF determination interfere with the experimental uncertainties and thus no overall theoretical uncertainty is quoted

$\alpha _\mathrm{s} (m_\mathrm{Z})$ values from H1 jet cross sections					
Data	$\tilde{\mu } _{\mathrm{cut}}$	$\alpha _\mathrm{s} (m_\mathrm{Z})$ with uncertainties	th	tot	$\chi ^{2}/n_\mathrm{dof}$
*Inclusive jets*					
$300\,\mathrm {GeV}$ high-$Q^{2}$	$2m_b$	$0.1221\,(31)_\mathrm{exp}\,(22)_\mathrm{had}\,(5)_\mathrm{PDF}\,(3)_\mathrm{PDF\alpha _\mathrm{s}}\,(4)_\mathrm{PDFset}\,(36)_\mathrm{scale}$	$(43)_\mathrm{th}$	$(53)_\mathrm{tot}$	6.5 / 15
HERA-I low-$Q^{2}$	$2m_b$	$0.1093\,(17)_\mathrm{exp}\,(8)_\mathrm{had}\,(5)_\mathrm{PDF}\,(5)_\mathrm{PDF\alpha _\mathrm{s}}\,(7)_\mathrm{PDFset}\,(33)_\mathrm{scale}$	$(35)_\mathrm{th}$	$(39)_\mathrm{tot}$	17.5 / 22
HERA-I high-$Q^{2}$	$2m_b$	$0.1136\,(24)_\mathrm{exp}\,(9)_\mathrm{had}\,(6)_\mathrm{PDF}\,(4)_\mathrm{PDF\alpha _\mathrm{s}}\,(4)_\mathrm{PDFset}\,(31)_\mathrm{scale}$	$(33)_\mathrm{th}$	$(41)_\mathrm{tot}$	14.7 / 23
HERA-II low-$Q^{2}$	$2m_b$	$0.1187\,(18)_\mathrm{exp}\,(8)_\mathrm{had}\,(4)_\mathrm{PDF}\,(4)_\mathrm{PDF\alpha _\mathrm{s}}\,(3)_\mathrm{PDFset}\,(45)_\mathrm{scale}$	$(46)_\mathrm{th}$	$(50)_\mathrm{tot}$	29.6 / 40
HERA-II high-$Q^{2}$	$2m_b$	$0.1121\,(18)_\mathrm{exp}\,(9)_\mathrm{had}\,(5)_\mathrm{PDF}\,(4)_\mathrm{PDF\alpha _\mathrm{s}}\,(2)_\mathrm{PDFset}\,(35)_\mathrm{scale}$	$(37)_\mathrm{th}$	$(41)_\mathrm{tot}$	42.5 / 29
*Dijets*					
$300\,\mathrm {GeV}$ high-$Q^{2}$	$2m_b$	$0.1213\,(39)_\mathrm{exp}\,(17)_\mathrm{had}\,(5)_\mathrm{PDF}\,(2)_\mathrm{PDF\alpha _\mathrm{s}}\,(3)_\mathrm{PDFset}\,(31)_\mathrm{scale}$	$(35)_\mathrm{th}$	$(52)_\mathrm{tot}$	13.6 / 15
HERA-I low-$Q^{2}$	$2m_b$	$0.1101\,(23)_\mathrm{exp}\,(8)_\mathrm{had}\,(5)_\mathrm{PDF}\,(4)_\mathrm{PDF\alpha _\mathrm{s}}\,(5)_\mathrm{PDFset}\,(36)_\mathrm{scale}$	$(38)_\mathrm{th}$	$(45)_\mathrm{tot}$	10.4 / 20
HERA-II low-$Q^{2}$	$2m_b$	$0.1173\,(14)_\mathrm{exp}\,(9)_\mathrm{had}\,(5)_\mathrm{PDF}\,(5)_\mathrm{PDF\alpha _\mathrm{s}}\,(3)_\mathrm{PDFset}\,(44)_\mathrm{scale}$	$(45)_\mathrm{th}$	$(47)_\mathrm{tot}$	17.4 / 41
*Combined* $\alpha_s$ *fits*					
HERA-II high-$Q^{2}$	$2m_b$	$0.1089\,(21)_\mathrm{exp}\,(7)_\mathrm{had}\,(5)_\mathrm{PDF}\,(3)_\mathrm{PDF\alpha _\mathrm{s}}\,(3)_\mathrm{PDFset}\,(25)_\mathrm{scale}$	$(27)_\mathrm{th}$	$(34)_\mathrm{tot}$	28.0 / 23
H1 inclusive jets	$2m_b$	$0.1132\,(10)_\mathrm{exp}\,(5)_\mathrm{had}\,(4)_\mathrm{PDF}\,(4)_\mathrm{PDF\alpha _\mathrm{s}}\,(2)_\mathrm{PDFset}\,(40)_\mathrm{scale}$	$(40)_\mathrm{th}$	$(42)_\mathrm{tot}$	134.0 / 133
H1 inclusive jets	$28\,\mathrm {GeV}$	$0.1152\,(20)_\mathrm{exp}\,(6)_\mathrm{had}\,(2)_\mathrm{PDF}\,(2)_\mathrm{PDF\alpha _\mathrm{s}}\,(3)_\mathrm{PDFset}\,(26)_\mathrm{scale}$	$(27)_\mathrm{th}$	$(33)_\mathrm{tot}$	44.1 / 60
H1 dijets	$2m_b$	$0.1148\,(11)_\mathrm{exp}\,(6)_\mathrm{had}\,(5)_\mathrm{PDF}\,(4)_\mathrm{PDF\alpha _\mathrm{s}}\,(4)_\mathrm{PDFset}\,(40)_\mathrm{scale}$	$(41)_\mathrm{th}$	$(42)_\mathrm{tot}$	93.9 / 102
H1 dijets	$28\,\mathrm {GeV}$	$0.1147\,(24)_\mathrm{exp}\,(5)_\mathrm{had}\,(3)_\mathrm{PDF}\,(2)_\mathrm{PDF\alpha _\mathrm{s}}\,(3)_\mathrm{PDFset}\,(24)_\mathrm{scale}$	$(25)_\mathrm{th}$	$(35)_\mathrm{tot}$	30.8 / 43
H1 jets	$2m_b$	$0.1143\,(9)_\mathrm{exp}\,(6)_\mathrm{had}\,(5)_\mathrm{PDF}\,(5)_\mathrm{PDF\alpha _\mathrm{s}}\,(4)_\mathrm{PDFset}\,(42)_\mathrm{scale}$	$(43)_\mathrm{th}$	$(44)_\mathrm{tot}$	195.0 / 199
H1 jets	$28\,\mathrm {GeV}$	$0.1157\,(20)_\mathrm{exp}\,(6)_\mathrm{had}\,(3)_\mathrm{PDF}\,(2)_\mathrm{PDF\alpha _\mathrm{s}}\,(3)_\mathrm{PDFset}\,(27)_\mathrm{scale}$	$(28)_\mathrm{th}$	$(34)_\mathrm{tot}$	63.2 / 90
H1 jets	$42\,\mathrm {GeV}$	$0.1168\,(22)_\mathrm{exp}\,(7)_\mathrm{had}\,(2)_\mathrm{PDF}\,(2)_\mathrm{PDF\alpha _\mathrm{s}}\,(5)_\mathrm{PDFset}\,(17)_\mathrm{scale}$	$(20)_\mathrm{th}$	$(30)_\mathrm{tot}$	37.6 / 40
*PDF+ * $\alpha_s$ *fits*					
	$2m_b$	$0.1142\,(11)_\mathrm{exp,NP,PDF}\,(2)_\mathrm{mod}\,(2)_\mathrm{par}\,(26)_\mathrm{scale}$		$(28)_\mathrm{tot}$	1539.7 / 1516

For the fits to the individual data sets the $\chi ^{2}$/$n_\mathrm{dof}$ is around unity in most cases. The $\alpha _\mathrm{s} (m_\mathrm{Z})$ values are all found to be consistent, in particular between inclusive jet and dijet measurements.

The fits to the inclusive jet data exhibit $\chi ^{2}$/$n_\mathrm{dof}$ values around unity, thus indicating the consistency of the individual data sets. The value of $\alpha _\mathrm{s} (m_\mathrm{Z})$ from ‘H1 inclusive jets’ has a significantly reduced experimental uncertainty compared to the results for the individual data sets. The cut $\tilde{\mu } >28\,\mathrm {GeV}$ results for inclusive jets in $\alpha _\mathrm{s} (m_\mathrm{Z}) =0.1152\,(20)_\mathrm{exp}\,(27)_\mathrm{th}$, which is consistent with the world average [2, 108].

Value of $\chi ^{2}$/$n_\mathrm{dof}$ around unity are obtained for fits to all dijet cross sections confirming their consistency. The results agree with those from inclusive jet cross sections and the world average. At high scales $\tilde{\mu } >28\,\mathrm {GeV}$, a value $\alpha _\mathrm{s} (m_\mathrm{Z}) =0.1147\,(24)_\mathrm{exp}\,(25)_\mathrm{th}$ is found.

The fit to H1 jets yields $\chi ^{2}/n_\mathrm{dof}= 0.98$ for 200 data points and $\alpha _\mathrm{s} (m_\mathrm{Z}) =0.1143\,(9)_\mathrm{exp}\,(43)_\mathrm{th}$. The scale uncertainty is the largest among the theoretical uncertainties and all other uncertainties are negligible in comparison.

The $\alpha _\mathrm{s} (m_\mathrm{Z})$ value obtained from H1 jet data restricted to $\tilde{\mu } >28\,\mathrm {GeV}$ is

$$\begin{aligned} \alpha _\mathrm{s} (m_\mathrm{Z})&= 0.1157\,(20)_\mathrm{exp}\,(6)_\mathrm{had}\,(3)_{\mathrm{PDF}}\,(2)_\mathrm{PDF\alpha _\mathrm{s}}\\&\quad (3)_{\mathrm{PDFset}}\,(27)_{\mathrm{scale}} \end{aligned}$$

with $\chi ^{2}= 63.2$ for 91 data points. Although the reduced number of data points leads to an increased experimental uncertainty, as compared to the option $\tilde{\mu } >2m_b$, it is still smaller than the scale uncertainty, which is found to be reduced significantly. All PDF related uncertainties essentially vanish.^1^ Therefore, this $\alpha _\mathrm{s} (m_\mathrm{Z})$ determination is taken as the main result. This result as well as those results obtained from the inclusive jet and dijet data separately are consistent with the world average.

The main result is also found to be consistent with $\alpha _\mathrm{s} (m_\mathrm{Z}) =0.1165(8)_{\text {exp}}(38)_{\text {pdf,theo}}$ determined previously in NLO accuracy from normalised H1 HERA-II high-$Q^{2}$ jet cross section data [24]. That result is experimentally more precise, mainly because data at somewhat lower scales and three-jet data are included.^2^ The scale uncertainty of the previous NLO fit is larger than for the present analysis in NNLO, despite of the fact that it was considered to be partially uncorrelated bin-to-bin in the previous NLO fit, whereas the present approach is more conservative.

In the present analysis, the value with the smallest total uncertainty is obtained in a fit to H1 jets restricted to $\tilde{\mu } >42\,\mathrm {GeV}$ with the result $\alpha _\mathrm{s} (m_\mathrm{Z}) =0.1168\,(22)_\mathrm{exp}\,(20)_\mathrm{theo}$ and a value of $\chi ^{2}/n_\mathrm{dof}=37.6/40$. This result, however, is obtained from a very limited number of measurements, the precision of which is limited by statistical uncertainties.

The ratio of all H1 jet cross section measurements to the NNLO predictions is displayed in Fig. 12. Overall good agreement between data and predictions is observed.

*Running of the strong coupling constant* The strong coupling is determined in fits to data points grouped into intervals $[\tilde{\mu } _\mathrm{lo};\tilde{\mu } _\mathrm{up}]$ of $\tilde{\mu }$. The data point grouping and the interval boundaries can be read off Fig. 12. The assumptions on the running of $\alpha _\mathrm{s}(\mu _\mathrm{R})$ thus are for each fit restricted to a limited $\mu _\mathrm{R}$ range.^3^ For a given data point its $\tilde{\mu }$ value is representative for the $\mu _\mathrm{R}$ range probed by the corresponding prediction, see Eqs. (8) and (9). The fit results are for each interval shown at the representative scale $\mu _\mathrm{R} =\sqrt{\tilde{\mu } _\mathrm{lo}\tilde{\mu } _\mathrm{up}}$.

The results for fits to inclusive jet and to dijet cross sections, as well as to H1 jets, are presented for the ten selected intervals in $\tilde{\mu }$ in Table 5 and are displayed in Fig. 13. Consistency is found for the fits to inclusive jets, dijets, and H1 jets, and the running of the strong coupling is confirmed in the accessible range of approximately 7 to $90\,\mathrm {GeV}$. The lowest interval considered contains the data points with $\tilde{\mu } <2m_b$, which are excluded from the main analysis. Nevertheless, these results are found to be consistent with the other $\alpha _\mathrm{s} (m_\mathrm{Z})$ determinations presented here.

**Fig. 27:**
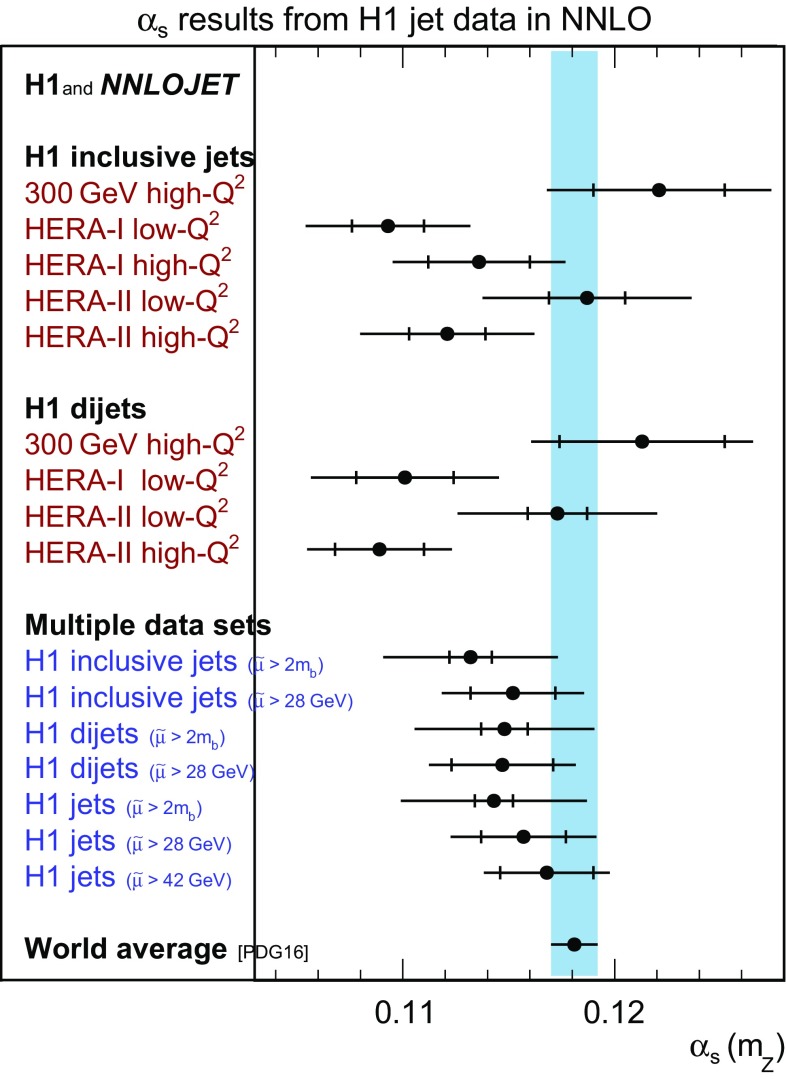
Summary of $\alpha _\mathrm{s} (m_\mathrm{Z})$ values obtained from fits to individual and multiple H1 jet data sets. The inner error bars indicate the experimental uncertainty and the outer error bars the total uncertainty

The values obtained from fits to H1 jets are compared to other determinations of at least NNLO accuracy [41, 44, 54, 109] and to results at NLO at very high scale [52] in Fig. 14, and consistency with the other experiments is found.

The results are consistent with results obtained from an alternative method used as a cross check, where in a single fit with ten free parameters the $\alpha _\mathrm{s}$ values in the ten bins are determined simultaneously.

## Simultaneous ${\varvec{\alpha _\mathrm{s}}}$ and PDF determination

In addition to the fits described above also a fit in NNLO accuracy of $\alpha _\mathrm{s} (m_\mathrm{Z})$ together with the non-perturbative PDFs is performed which takes jet data and inclusive DIS data as input. This fit is denoted as ‘PDF+$\alpha _\mathrm{s}$-fit’ in the following.

### Methodology

The methodology of the PDF+$\alpha _\mathrm{s}$-fit is closely related to PDF determinations as performed by other groups [56, 58, 82, 104, 105]. The PDFs are parametrised at a low starting scale $\mu _0$ which is below the charm-quark mass. Heavy-quark PDFs are generated dynamically and only light-quark PDFs and the gluon distribution have to be determined in the fit.

In order to have constraints on the PDFs, polarised and unpolarised inclusive NC and CC DIS cross sections [61–66] are used (Table 3). This data sample is identical to the one used in the H1PDF2012 PDF fit [65]. In addition, normalised inclusive jet and dijet cross sections [15, 21, 24] are used (Table 2).

The calculations of the splitting kernels are performed in NNLO using the program QCDNUM [96, 97]. The predictions for the inclusive DIS cross sections are calculated using structure function calculations in NNLO using the zero-mass variable flavour number scheme (ZM-VFNS) [65] as implemented in QCDNUM [96, 97]. Normalised jet cross sections are calculated as a ratio of jet cross sections to inclusive NC DIS, where the former are calculated as outlined in Sect. 3.1 and the latter are calculated using ZM-VFNS structure functions using QCDNUM. For inclusive DIS predictions the scales $\mu _\mathrm{R} ^2$ and $\mu _\mathrm{F} ^2$ are both set to $Q^{2}$ and for jet predictions to $Q^{2}+P_{T}^2$, as specified in Eq. (6).

For the PDF+$\alpha _\mathrm{s}$-fit all data are restricted to the range $Q^{2}>10\,\mathrm {GeV}^2$ in order to exclude kinematic regions where fixed-order pQCD cannot be applied reliably. For jet cross sections $\tilde{\mu } >2m_b$ is required in addition. After applying these cuts, the jet predictions receive contributions from the *x*-range down to 0.003, whereas without these cuts it would be 0.002. Major contributions to the data points at highest values of $\tilde{\mu }$ are within the *x*-range 0.1 to 0.5.

**Fig. 28:**
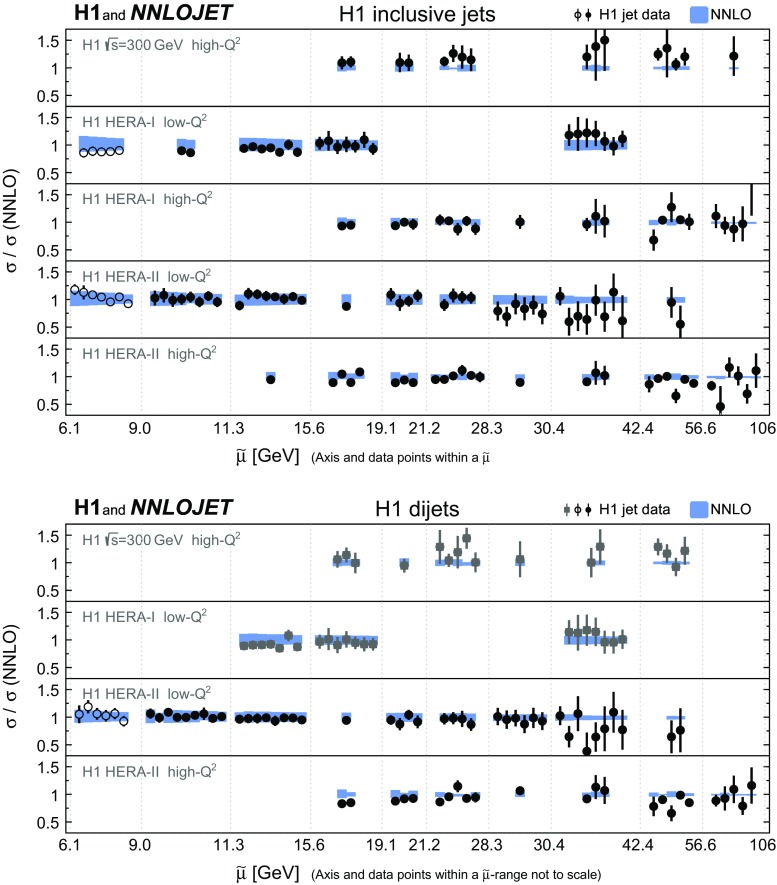
Ratio of inclusive jet (upper panel) and dijet cross sections (lower panel) to NNLO predictions obtained with the fitted value $\alpha _\mathrm{s} (m_\mathrm{Z}) =0.1157$. Data points are ordered according to their scale $\tilde{\mu }$ and are displayed on the horizontal axis within the respective $\tilde{\mu }$-interval. Within a single interval multiple data points are displayed with equal horizontal spacing and are thus not to scale. The displayed intervals reflect the choices made for the studies of the running of the strong coupling (compare Figs. 13 and 14). The shaded area indicates the uncertainty on the NNLO calculations from scale variations. The open circles show data points which are not considered for some fits, because their scale $\tilde{\mu }$ is below $2m_b$. The squares show data points not considered for the ‘H1 jets’-fit, since the statistical correlations to the respective inclusive jet measurements are not known

The choice of the PDF parametrisations and the values of input parameters follows closely previous approaches [56, 63, 65, 110] and are only discussed briefly here. At a starting scale $\mu _0^2=1.9\,\mathrm {GeV}^2$ parton densities are attributed to the constituents of the proton. These take the functional form

11 $$\begin{aligned} xf(x)|_{\mu _{0}} = f_Ax^{f_B}(1-x)^{f_C}(1+f_Dx+f_Ex^2), \end{aligned}$$

where *f* is one of *g*, $\tilde{u}$, $\tilde{d}$, $\bar{U}$, $\bar{D}$, denoting the density of the gluon, up-valence, down-valence, up-sea, down-sea in the proton, respectively. The strange sea is set to $\bar{s}(x)=f_s\bar{D}$, where $f_s=0.4$. Parameters $f_D$ and $f_E$ are set to zero by default, but are added for specific flavours in order to improve the fit. The parameters $g_A$, $\tilde{u}_A$ and $\tilde{d}_A$ are constrained by sum rules. The parameter $\bar{U}_A$ is set equal to $\bar{D}_A(1-f_s)$. The parameter $\bar{U}_B$ is set equal to $\bar{D}_B$. A total of 12 fit parameters are used to describe the PDFs.

**Table 5:** Values of the strong coupling constant $\alpha _\mathrm{s}(\mu _\mathrm{R})$ and at the *Z*-boson mass, $\alpha _\mathrm{s} (m_\mathrm{Z})$, obtained from fits to groups of data points with comparable values of $\mu _\mathrm{R}$. The first (second) uncertainty of each point corresponds to the experimental (theory) uncertainty. The theory uncertainties include PDF related uncertainties and the dominating scale uncertainty

Running of the strong coupling						
$\mu _\mathrm{R}$ $[\hbox {GeV}]$	Inclusive jets		Dijets		H1 jets	
	$\alpha _\mathrm{s} (m_\mathrm{Z})$	$\alpha _\mathrm{s}(\mu _\mathrm{R})$	$\alpha _\mathrm{s} (m_\mathrm{Z})$	$\alpha _\mathrm{s}(\mu _\mathrm{R})$	$\alpha _\mathrm{s} (m_\mathrm{Z})$	$\alpha _\mathrm{s}(\mu _\mathrm{R})$
7.4	$0.1148\,(13)\,(42)$	$0.1830\,(34)\,(114)$	$0.1182\,(28)\,(41)$	$0.1923\,(77)\,(116)$	$0.1147\,(13)\,(43)$	$0.1829\,(34)\,(114)$
10.1	$0.1136\,(17)\,(36)$	$0.1678\,(39)\,(81)$	$0.1169\,(14)\,(42)$	$0.1751\,(34)\,(99)$	$0.1148\,(14)\,(40)$	$0.1705\,(31)\,(91)$
13.3	$0.1147\,(15)\,(43)$	$0.1605\,(30)\,(88)$	$0.1131\,(18)\,(38)$	$0.1573\,(36)\,(76)$	$0.1144\,(15)\,(42)$	$0.1600\,(30)\,(86)$
17.2	$0.1130\,(15)\,(33)$	$0.1492\,(26)\,(59)$	$0.1104\,(19)\,(30)$	$0.1445\,(33)\,(53)$	$0.1127\,(15)\,(33)$	$0.1486\,(27)\,(59)$
20.1	$0.1136\,(17)\,(33)$	$0.1457\,(29)\,(56)$	$0.1116\,(22)\,(31)$	$0.1425\,(36)\,(52)$	$0.1134\,(17)\,(33)$	$0.1454\,(29)\,(55)$
24.5	$0.1173\,(17)\,(30)$	$0.1463\,(26)\,(48)$	$0.1147\,(23)\,(24)$	$0.1423\,(36)\,(38)$	$0.1171\,(17)\,(29)$	$0.1460\,(27)\,(46)$
29.3	$0.1084\,(36)\,(29)$	$0.1287\,(51)\,(41)$	$0.1163\,(34)\,(34)$	$0.1401\,(50)\,(50)$	$0.1134\,(30)\,(32)$	$0.1358\,(44)\,(46)$
36.0	$0.1153\,(32)\,(37)$	$0.1338\,(43)\,(50)$	$0.1135\,(37)\,(29)$	$0.1314\,(50)\,(39)$	$0.1146\,(30)\,(33)$	$0.1328\,(41)\,(44)$
49.0	$0.1170\,(22)\,(20)$	$0.1290\,(27)\,(25)$	$0.1127\,(31)\,(15)$	$0.1238\,(37)\,(18)$	$0.1169\,(23)\,(19)$	$0.1290\,(28)\,(24)$
77.5	$0.1111\,(55)\,(19)$	$0.1137\,(58)\,(20)$	$0.1074\,(84)\,(19)$	$0.1099\,(88)\,(20)$	$0.1113\,(55)\,(19)$	$0.1139\,(58)\,(20)$

The uncertainty obtained from the fit comprises experimental uncertainties of the data and hadronisation uncertainties of the jet cross section predictions. The resulting uncertainty of $\alpha _\mathrm{s} (m_\mathrm{Z})$ from the PDF+$\alpha _\mathrm{s}$-fit is denoted as ‘exp,had,PDF’. In order to determine also model (‘mod’) and parametrisation (‘par’) uncertainties, an additional error estimation similar to HERAPDF2.0 [56] is performed. The model uncertainty is estimated as the quadratic sum of the differences of the nominal result to the resulting values of $\alpha _\mathrm{s}$ when repeating the PDF+$\alpha _\mathrm{s}$-fit with alternative parameters, such as the charm or beauty masses or the sea quark suppression factor $f_\mathrm{s}$ [56]. Parametrisation uncertainties are attributed by adding extra $f_D$ or $f_E$ parameters to the fit or by varying the starting scale. In addition, a more flexible functional form is allowed for the gluon, similar to the PDF parametrisation used for the default HERAPDF2.0 [56] fit.^4^ A total of eight parametric forms different from the default are considered.

The scale uncertainty of $\alpha _\mathrm{s} (m_\mathrm{Z})$ from this fit is determined by repeating fits with scale factors 0.5 and 2 applied to $\mu _\mathrm{R}$ and $\mu _\mathrm{F}$ simultaneously to all calculations involved. The larger of the two deviations from the central fit, corresponding to a scale factor of 0.5, is taken as symmetric scale uncertainty. A more detailed study is beyond the scope of this paper.

The PDF+$\alpha _\mathrm{s}$-fit differs from the $\alpha _\mathrm{s}$-fit outlined in Sect. 3 in the following aspects: the usage of normalised jet cross sections, the inclusion of NC and CC DIS cross sections and the low starting scale $\mu _0$ of the DGLAP evolution, thus assuming the validity of the running coupling and the PDF evolution down to lower scale values.

### Results

*Fit results and the value of* ${{\alpha _\mathrm{s} (m_\mathrm{Z})}}$ The results of the PDF+$\alpha _\mathrm{s}$-fit are presented in Table 6. The fit yields $\chi ^{2}/n_\mathrm{dof}=1539.7/(1529-13)$, confirming good agreement between the predictions and the data. The resulting PDF is able to describe 141 jet data points and the inclusive DIS data simultaneously.

The value of $\alpha _\mathrm{s} (m_\mathrm{Z})$ is determined to

$$\begin{aligned} \alpha _\mathrm{s} (m_\mathrm{Z}) =0.1142\,(11)_\mathrm{exp,had,PDF}\,(2)_\mathrm{mod}\,(2)_\mathrm{par}\,(26)_\mathrm{scale}. \end{aligned}$$

and is determined to an overall precision of 2.5 %. It is worth noting that the result is largely insensitive to the PDF model and parametrisation choices. The scale uncertainty is dominating. The $\alpha _\mathrm{s} (m_\mathrm{Z})$ value is consistent with the main result of the ‘H1 jets’ fit. The result is compared to values from the PDF fitting groups ABM [111], ABMP [104], BBG [112], HERAPDF [56], JR [113], NNPDF [57] and MMHT [58] in Fig. 15 and consistency is found. The value is consistent with the world average and the ‘pre-average’ value of the structure function category [2]. The result exhibits a competitive experimental uncertainty to other determinations [57, 58, 104], which is achieved by using H1 normalised jet cross sections in addition to the H1 inclusive DIS data.

**Fig. 29:**
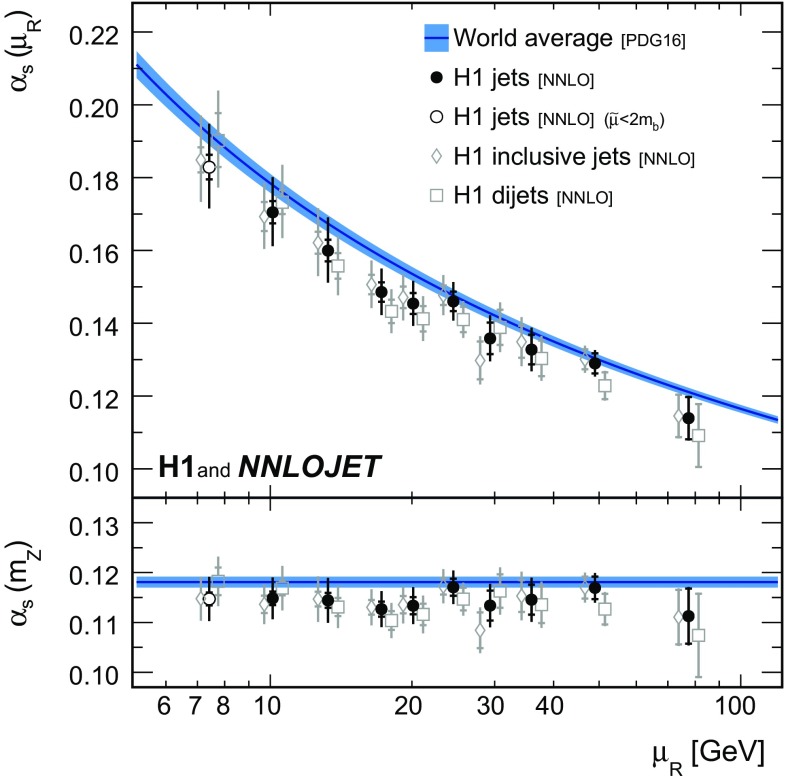
Results on $\alpha _\mathrm{s} (m_\mathrm{Z})$ and $\alpha _\mathrm{s}(\mu _\mathrm{R})$ for fits to data points arranged in groups of similar $\mu _\mathrm{R}$. The circles show results from inclusive jet and dijet data taken together (‘H1 jets’), the open diamonds results from inclusive jet cross sections alone and the open boxes results from dijet cross sections alone. For these fits, the data sets are not constrained by the requirement $\tilde{\mu } >2m_b$. The fitted values of $\alpha _\mathrm{s} (m_\mathrm{Z})$ (lower panel) are translated to $\alpha _\mathrm{s}(\mu _\mathrm{R})$ (upper panel), using the solution of the QCD renormalisation group equation. The data points from fits to inclusive jets (dijets) are displaced to the left (right) for better visibility. In the upper panel a displacement is also applied along the vertical direction, to account for the running of $\alpha _\mathrm{s}(\mu _\mathrm{R})$. The inner error bars denote the experimental uncertainties alone, and the outer error bars indicate the total uncertainties

**Fig. 30:**
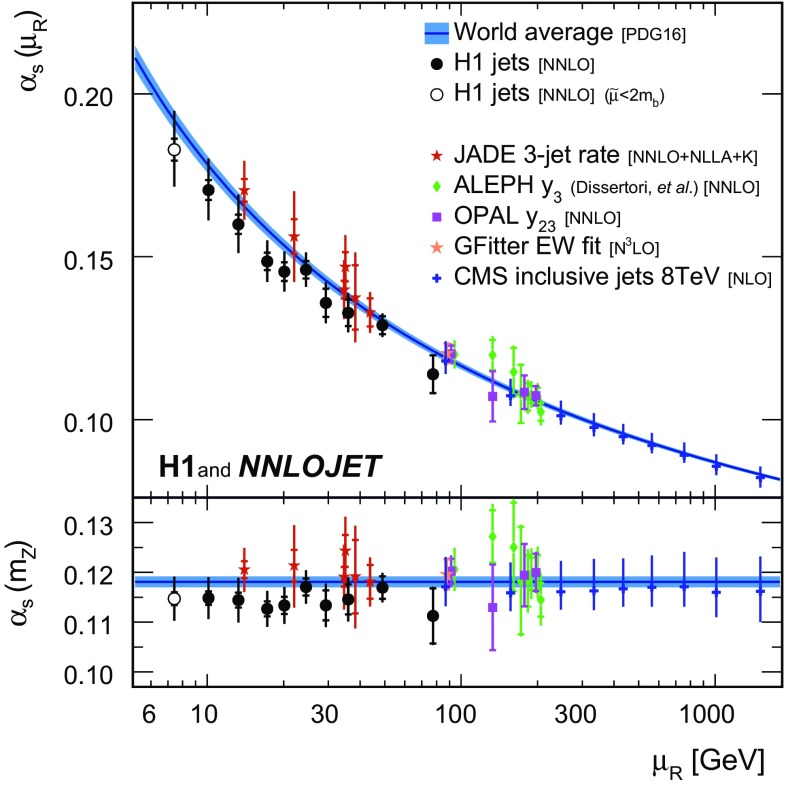
Results for $\alpha _\mathrm{s} (m_\mathrm{Z})$ and $\alpha _\mathrm{s}(\mu _\mathrm{R})$ for fits to data points arranged in groups of similar $\mu _\mathrm{R}$, compared to results from other experiments and processes. Further details can be found in the caption of Fig. 13

**Table 6:** Results of the PDF+$\alpha _\mathrm{s}$ fit. The columns denote the resulting fit value, its uncertainty and the correlations to the other parameters

Results for the PDF+${{{\alpha _\mathrm{s}}}}$-fit														
Parameter	Fit result	Correlation coefficients												
		$\alpha _\mathrm{s} (m_\mathrm{Z})$	$g_B$	$g_C$	$g_D$	$\tilde{u}_B$	$\tilde{u}_C$	$\tilde{u}_E$	$\tilde{d}_B$	$\tilde{d}_C$	$\bar{U}_C$	$\bar{D}_A$	$\bar{D}_B$	$\bar{D}_C$
$\alpha _\mathrm{s} (m_\mathrm{Z})$	$0.1142\pm 0.0011$	1												
$g_B$	$-0.023 \pm 0.035$	0.25	1											
$g_C$	$5.69 \pm 4.09$	$-0.08$	0.01	1										
$g_D$	$-0.44 \pm 4.20$	$-0.03$	$-0.10$	0.99	1									
$\tilde{u}_B$	$0.707 \pm 0.036$	0.39	0.25	0.05	0.04	1								
$\tilde{u}_C$	$4.909 \pm 0.096$	$-0.09$	$-0.13$	0.02	0.03	$-0.08$	1							
$\tilde{u}_E$	$12.7 \pm 1.8$	$-0.03$	$-0.25$	$-0.04$	$-0.01$	$-0.75$	0.57	1						
$\tilde{d}_B$	$1.036 \pm 0.098$	0.24	$-0.02$	0.06	0.08	0.32	$-0.24$	$-0.24$	1					
$\tilde{d}_C$	$5.35 \pm 0.49$	$-0.10$	$-0.07$	0.03	0.05	$-0.08$	$-0.24$	0.00	0.80	1				
$\bar{U}_C$	$4.96 \pm 0.86$	0.32	$-0.28$	$-0.01$	0.05	0.76	0.09	$-0.39$	0.53	0.11	1			
$\bar{D}_A$	$0.299 \pm 0.032$	0.29	$-0.71$	$-0.04$	0.07	0.32	0.01	$-0.08$	0.38	0.13	0.71	1		
$\bar{D}_B$	$-0.091 \pm 0.017$	0.22	$-0.79$	$-0.05$	0.06	0.19	0.03	0.01	0.29	0.09	0.61	0.97	1	
$\bar{D}_C$	$16.1 \pm 3.8$	$-0.13$	$-0.51$	$-0.01$	0.08	$-0.15$	$-0.24$	$-0.06$	0.14	0.24	0.05	0.48	0.46	1
$g_A$	2.84	Constrained by sum-rules												
$\tilde{u}_A$	4.11	Constrained by sum-rules												
$\tilde{d}_A$	6.94	Constrained by sum-rules												
$\bar{U}_A$	1.80	Set equal to $\bar{D}_A(1-f_s)$												
$\bar{U}_B$	$-0.091$	Set equal to $\bar{D}_B$												

**Fig. 31:**
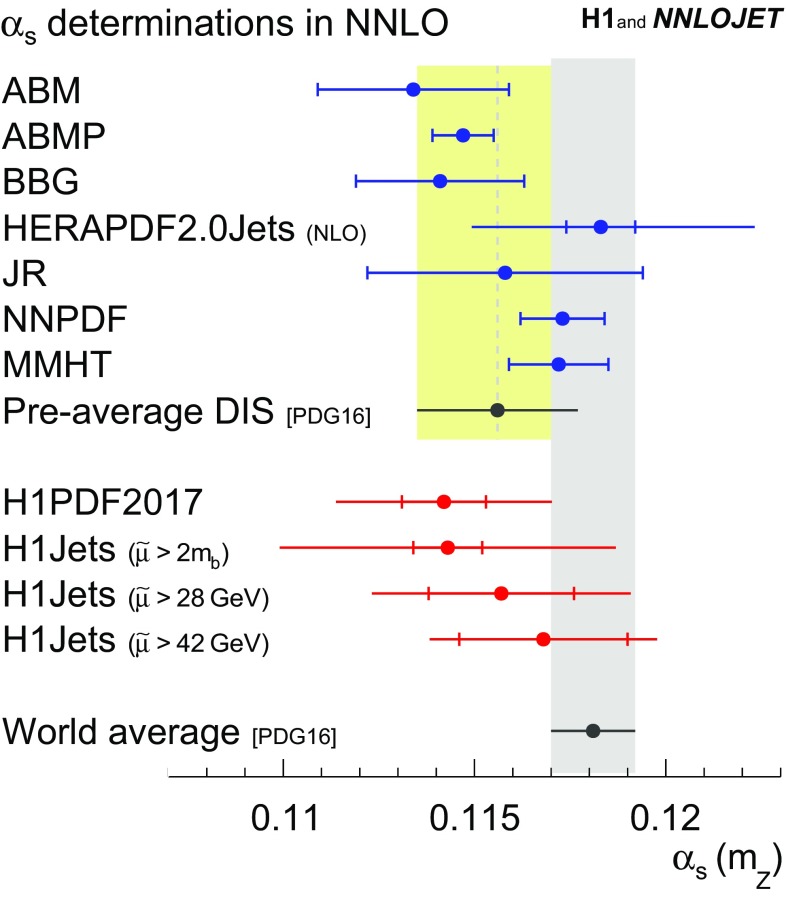
Comparison of the value of $\alpha _\mathrm{s} (m_\mathrm{Z})$ obtained in the  PDF+$\alpha _\mathrm{s}$-fit and in the H1 jets fit in NNLO accuracy to other $\alpha _\mathrm{s}$ determinations from DIS data. The pre-average of structure function data and the world average [2] are also indicated

*PDF parametrisation results* The PDF and $\alpha _\mathrm{s} (m_\mathrm{Z})$ parameters determined together in this fit (Table 6) are denoted as . It is released [114] in the LHAPDF [93] format with experimental, hadronisation and $\alpha _\mathrm{s} (m_\mathrm{Z})$ uncertainties included. The gluon and singlet momentum distributions, *xg* and $x\Sigma$, the latter defined as the sum of all quark and anti-quark densities, are compared to NNPDF3.1 at a scale $\mu _\mathrm{F} =20\,\mathrm {GeV}$ in Fig. 16. The uncertainties of the fitted PDFs are somewhat larger than the uncertainties of NNPDF3.1. For NNPDF3.1, $\alpha _\mathrm{s} ^\mathrm{PDF}(m_\mathrm{Z})$ is fixed while it is a free parameter in the  fit. Within uncertainties, the singlet distribution obtained for  is in fair agreement with NNPDF3.1 over a large range in *x*, whereas the gluon density is consistent with NNPDF3.1 only for $x>0.01$ and is significantly higher than NNPDF3.1 at lower *x*. This difference can not be explained by the assumptions made on the strong coupling in NNPDF3.1, as can be seen from the NNPDF3.1 distributions obtained for $\alpha _\mathrm{s} ^\mathrm{PDF}(m_\mathrm{Z}) =0.114$. However, there are differences in the datasets used for the fits. For  only H1 data are considered, restricted to the range $Q^2>10\,\text {GeV}^2$. For NNPDF3.1 the combined HERA DIS data [56] are used, starting from $Q^2>3.5\,\text {GeV}^2$. Data from other processes and experiments are also included, but no DIS jet data.

**Fig. 32:**
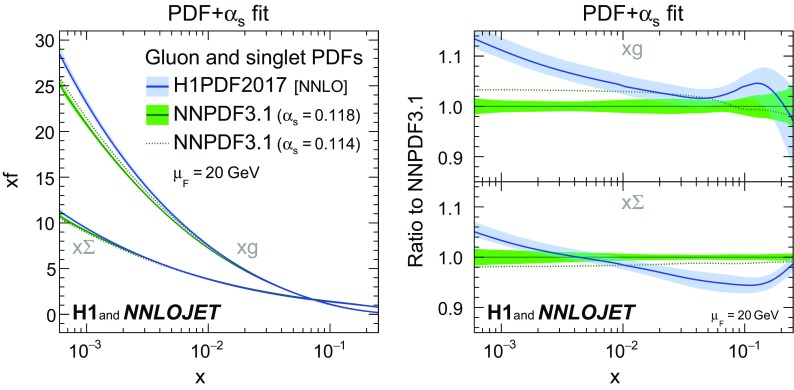
Gluon and singlet distributions determined by the PDF+$\alpha _\mathrm{s}$-fit, denoted as , as a function of the convolution variable *x* (see Eq. (1)). The distributions are displayed at $\mu _\mathrm{F} =20\,\mathrm {GeV}$. The PDFs are compared to the NNPDF3.1 PDFs determined with values of $\alpha _\mathrm{s} ^\mathrm{PDF}(m_\mathrm{Z})$ of 0.114 and 0.118. Ratios to NNPDF3.1 are shown in the right panels

The PDFs obtained for each of the model and parametrisation variations (not shown in Fig. 16) are contained in the exp,had,PDF uncertainty band for $x>0.0004$ and thus do not explain the differences to NNDPF3.1.

*The impact of H1 jet data on PDF fits* The PDF+$\alpha _\mathrm{s}$-fit is repeated with the normalised jet data excluded, i.e. only inclusive DIS data are considered. For this fit and the  fit the gluon distribution $xg(x,\mu _\mathrm{F})$ is evaluated at $\mu _\mathrm{F} =20\,\mathrm {GeV}$ and $x=0.01$ and its Hessian uncertainty together with its correlation coefficient with $\alpha _\mathrm{s} (m_\mathrm{Z})$ are calculated. The resulting Hessian error ellipses are displayed in Fig. 17 at a confidence level of $68\,\%$. Compared to the fit without jet data, the inclusion of jet data significantly reduces the uncertainties of $\alpha _\mathrm{s} (m_\mathrm{Z})$ and *xg*, as well as their correlation. The correlation coefficient is −0.92 and reduces to −0.65 if jet data are included. Also shown is the gluon distribution of NNPDF3.1 determined for different values of $\alpha _\mathrm{s} ^\mathrm{PDF}(m_\mathrm{Z})$. At this particular choice of *x* and $\mu _\mathrm{F}$, the gluon density of  is found to be consistent with NNPDF3.1 in the range where $\alpha _\mathrm{s} ^\mathrm{PDF}(m_\mathrm{Z})$ is close to the result of the  fit.

The two fits are repeated for each of the model and parametrisation variations (not shown in Fig. 17). For the  fit, only small variations of the results are observed, in accord with the small model and parametrisation uncertainties assigned to $\alpha _\mathrm{s} (m_\mathrm{Z})$ . However, if the jet data are not included in the fit, the resulting $\alpha _\mathrm{s} (m_\mathrm{Z})$ and *xg* are found to be strongly dependent on the assumptions made for the PDF parametrisation. This confirms previous observations [115], namely that *xg* and $\alpha _\mathrm{s} (m_\mathrm{Z})$ together cannot be determined reliably from H1 inclusive DIS data alone.

In summary, the inclusion of jet data allows for a reliable determination of $\alpha _\mathrm{s} (m_\mathrm{Z})$ and its uncertainty. It also stabilises the gluon density determination. In contrast to a previous study using only a fraction of the H1 data [17], it can now be stated that all H1 jet data taken together with all H1 inclusive DIS data do allow for a simultaneous determination of *xg* and $\alpha _\mathrm{s} (m_\mathrm{Z})$, with a precision on *xg* competitive to global PDF fits obtained using fixed value of $\alpha _\mathrm{s} ^\mathrm{PDF}(m_\mathrm{Z})$.

**Fig. 33:**
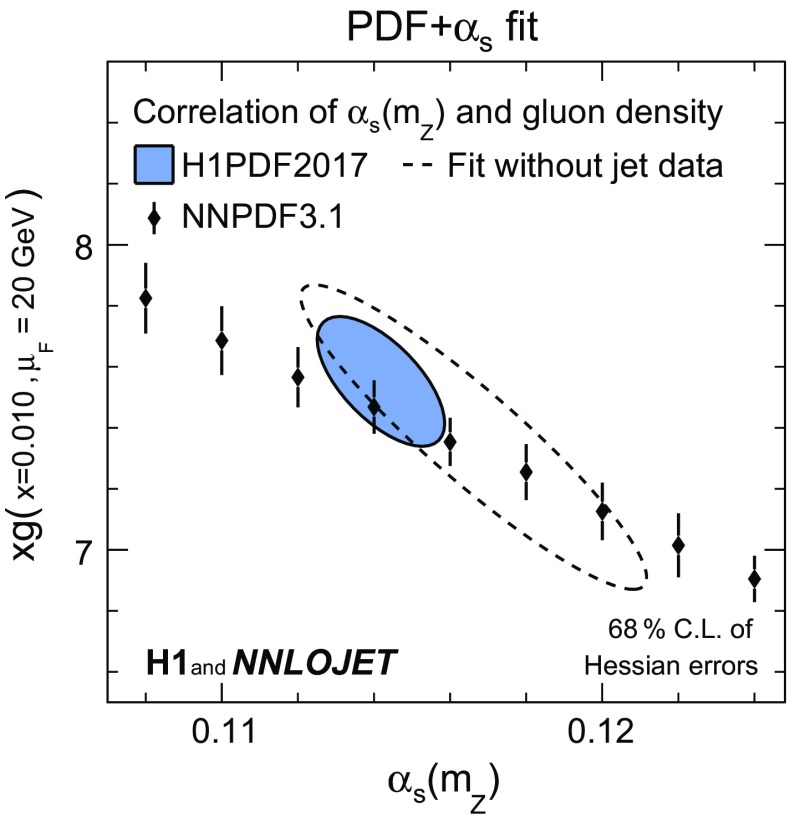
Error ellipses of Hessian uncertainties at $68\,\%$ confidence level of $\alpha _\mathrm{s} (m_\mathrm{Z})$ and the gluon density *xg* at $\mu _\mathrm{F} =20\,\mathrm {GeV}$ and $x=0.01$ as a result of two different PDF+$\alpha _\mathrm{s}$-fits. The filled ellipse indicates the result of the  fit and the dashed line of a PDF+$\alpha _\mathrm{s}$-fit with jet data excluded. The error ellipses represent the combined effect of experimental and hadronisation uncertainties as described in the text. The diamonds indicate the gluon density of the NNPDF3.1 PDF set for fixed values $\alpha _\mathrm{s} ^\mathrm{PDF}(m_\mathrm{Z})$

## Summary

The new next-to-next-to-leading order pQCD calculations (NNLO) for jet production cross sections in neutral-current DIS are exploited for a determination of the strong coupling constant $\alpha _\mathrm{s} (m_\mathrm{Z})$ using inclusive jet and dijet cross section measurements published by the H1 collaboration. Two methods are explored to determine the value of $\alpha _\mathrm{s} (m_\mathrm{Z})$.

In the first approach H1 inclusive jet and dijet data are analysed. The cross section predictions account for the $\alpha _\mathrm{s}$ dependence in the two components of the calculations, the partonic cross sections and the parton distribution functions (PDFs). The strong coupling constant is determined to be $\alpha _\mathrm{s} (m_\mathrm{Z}) =0.1157\,(20)_\mathrm{exp}\,(29)_\mathrm{th}$, where the jet data are restricted to high scales $\tilde{\mu } > 28\,\mathrm {GeV}$. Uncertainties due to the input PDFs or the hadronisation corrections are found to be small, and the largest source of uncertainty is from scale variations of the NNLO calculations. The experimental uncertainty may be reduced to 0.8 %, if all inclusive jet and dijet data with $\tilde{\mu } >2m_b$ are considered, but the scale uncertainties are increased significantly. The smallest total uncertainty on $\alpha _\mathrm{s} (m_\mathrm{Z})$ of 2.5 % is obtained when restricting the data to $\tilde{\mu } >42\,\mathrm {GeV}$. Values of $\alpha _\mathrm{s} (m_\mathrm{Z})$ determined from inclusive jet data or dijet data alone are found to be consistent with the main result. All these results are found to be consistent with each other and with the world average value of $\alpha _\mathrm{s} (m_\mathrm{Z})$.

The running of the strong coupling constant is tested in the range of approximately 7 to $90\,\mathrm {GeV}$ by dividing the jet data into ten subsets of approximately constant scale. The scale dependence of the coupling is found to be consistent with the expectation.

In a second approach a combined determination of PDF parameters and $\alpha _\mathrm{s} (m_\mathrm{Z})$ in NNLO accuracy is performed. In this fit all normalised inclusive jet and dijet cross sections published by H1 are analysed together with all inclusive neutral-current and charged-current DIS cross sections determined by H1. Using the data with $Q^{2}>10\,\mathrm {GeV}^2$, the value of $\alpha _\mathrm{s} (m_\mathrm{Z})$ is determined to be $\alpha _\mathrm{s} (m_\mathrm{Z}) =0.1142\,(28)_\mathrm{tot}$. Consistency with the other results and the world average is found. The resulting PDF set  is found to be consistent with the NNPDF3.1 PDF set at sufficiently large $x>0.01$, albeit there are differences at lower *x*. It is demonstrated that the inclusion of H1 jet data into such a simultaneous PDF and $\alpha _\mathrm{s} (m_\mathrm{Z})$ determination provides stringent constraints on $\alpha _\mathrm{s} (m_\mathrm{Z})$ and the gluon density. The results and their uncertainties are found to be largely insensitive to the assumptions made for the PDF parametrisation.

Relevant phenomenological aspects of the NNLO calculations are studied for the first time. The NNLO calculations are repeated for a number of different scale choices and scale factors, as well as for a large variety of recent PDF sets. The level of agreement with H1 jet data is judged quantitatively. The NNLO calculations improve significantly the description of the data and reduce the dominating theoretical uncertainty on $\alpha _\mathrm{s} (m_\mathrm{Z})$ in comparison to previously employed NLO calculations. All jet cross section measurements are found to be well described by the NNLO predictions. These NNLO calculations are employed for a PDF determination for the first time.

This is the first precision extraction of $\alpha _\mathrm{s} (m_\mathrm{Z})$ from jet data at NNLO involving a hadron in the initial state. It opens a new chapter of precision QCD measurements at hadron colliders.

## Footnotes

1 The difference of the main fit result to $\alpha _\mathrm{s} ^\mathrm{PDF}(m_\mathrm{Z}) =0.118$ is covered by the systematic variation $\alpha _\mathrm{s} ^\mathrm{PDF}(m_\mathrm{Z}) =0.118\pm 0.002$.

2 No NNLO calculation is available for three-jet production in DIS to date.

3 For purely technical reasons the fit parameter is $\alpha _\mathrm{s} (m_\mathrm{Z})$, and thus the running is applied from $\mu _\mathrm{R}$, as used in the calculation, to $m_\mathrm{Z}$ and then ‘back’ to a representative value $\mu _\mathrm{R}$ .

4 The functional form referred to as “alternative gluon” in [56] actually corresponds to the default choice for this paper.
